# Bone marrow mesenchymal stem cells are functionally remodeled after complete remission of acute myeloid leukemia

**DOI:** 10.1515/jtim-2026-0048

**Published:** 2026-08-24

**Authors:** Zhiwei Zhang, Zhenkun Wang, Xinyan Zhang, Dandan Chen, Chenyuan Li, Lijuan Hu, Zhengli Xu, Xiaojun Huang, Yuan Kong

**Affiliations:** Peking University People’s Hospital, Peking University Institute of Hematology, National Clinical Research Center for Hematologic Disease, Beijing Key Laboratory of Cell and Gene Therapy for Hematologic Malignancies, Peking University, Beijing, China; Peking-Tsinghua Center for Life Sciences, Academy for Advanced Interdisciplinary Studies, Peking University, Beijing, China

**Keywords:** hematopoiesis, mesenchymal stem cells, acute myeloid leukemia, remodel, cellular senescence, chemo-therapy

## Abstract

**Background and Objectives:**

Acute myeloid leukemia (AML) is a type of hematological malignancy, induces the normal bone marrow (BM) microenvironment to supporting AML cells. Although previous work revealed that functional remodeling of BM endothelial progenitor cells after complete remission (CR), as another important component of the BM microenvironment, whether AML-induced BM mesenchymal stem cells (MSCs) can also be functional modified after CR is largely unknown.

**Methods:**

Patients with de novo AML, patients with AML who achieved complete remission (AML-CR) and healthy controls (HC) were enrolled. Flow cytometry was used to exploring the regulatory effects of BM MSCs on cluster of differentiation CD34^+^ cells or CD3^+^ cells. Immunocytochemistry and senescence-associated β-galactosidase (SA-β-gal) staining was used to detect the number of senescent BM MSCs. Ribonucleic acid sequencing (RNA-seq) analysis and quantitative real-time reverse transcription PCR (qRT-PCR) were used to detect the transcription profile and characteristics of BM MSCs. An AML-CR mouse model was used to verify the remodeling of BM MSCs after CR.

**Results:**

Dysfunctional BM MSCs in AML patients were subsequently recovered in AML-CR patients, including their differentiation potential, apoptosis ratio, expression of cytokines and regulatory roles, which were return to near HC MSCs. Moreover, the transcriptomic profile of CR MSCs was also remodeled and transformed to be more similar to that of HC MSCs. Our data further revealed that the expression of senescent-related genes, the number of senescent cells in AML-CR patients were reduced, and that CR MSCs regained the ability to be reinduced to senescence by AML cells.

**Conclusions:**

Our findings describe the functional remodeling of BM MSCs among patients with AML, AML-CR patients and HC, and the reduction in the number of senescent BM MSCs associated with BM MSC remodeling. Our work provides a new perspective indicating that AML-modified BM MSCs can be remodeled to support normal hematopoietic stem cells (HSCs) in AML-CR patients, which may contribute to promoting normal hematopoiesis recovery for AML patients.

## Introduction

Normal hematopoiesis is maintained by hematopoietic stem cells (HSCs), and HSC homeostasis is stringently regulated by the bone marrow (BM) microenvironment.^[1,2]^ Acute myeloid leukemia (AML) is a kind of hematological malignancy characterized by aberrant and uncontrolled HSC proliferation, accompanied by an abnormal BM microenvironment, and seriously endangers human health.^[3, 4, 5, 6, 7]^ AML cells can create a BM microenvironment conducive to their own growth.^[8]^ Our previous work demonstrated that BM endothelial cells, as one of the main components of the BM microenvironment, exhibit functional abnormalities during de novo AML, and that these dysfunctions are remodeled when patients achieve complete remission (CR), with endothelial cells becoming more similar to normal.^[9]^ As for another critical component of the BM microenvironment, accumulating evidence indicates that mesenchymal stem cells (MSCs) exhibit various functional and phenotypic alterations and contribute to AML progression.^[10,11]^ However, whether MSCs also undergo functional recovery after chemotherapy-induced CR remains to be investigated.

MSCs support immunoregulation and HSC maintenance through the secretion of cytokines, cell-cell interactions, and a tuned balance of self-renewal and differentiation.^[12, 13, 14]^ MSCs are heterogeneous, can be induced to support tumor cell growth, and play major pathogenic roles in several hematopoietic malignancies.^[15, 16, 17, 18]^ In the context of AML, BM MSCs interact with leukemic cells through multiple mechanisms to promote disease progression. Emerging evidence has shown that BM MSCs support the survival, proliferation, and drug resistance of AML cells by activating antiapoptotic pathways via direct cell-cell contact or the secretion of cytokines (*i.e*., IL-6, CXCL12, VEGF, and SCF).^[13,19,20]^ In addition, BM MSCs suppress the homing, survival, and expansion of normal HSCs.^[21]^ Moreover, BM MSCs contribute to immune evasion of AML cells by upregulating the expression of immunosuppressive molecules and modulating antigen presentation processes.^[22]^ However, the functional status of BM MSCs after CR remains poorly understood. Therefore, investigating whether the function of BM MSCs is remodeled after CR, as well as elucidating the differences in MSC states among patients with de novo AML, patients with CR and HC, is highly important for promoting the restoration of normal hematopoiesis in patients with AML.

It has been well studied MSCs exhibit various functional and phenotypic alterations in the context of AML. For example, compared with normal MSCs, AML-associated MSCs display reduced osteogenic differentiation potential, enhanced adipogenic differentiation capacity, and a diminished ability to support normal HSCs.^[22,23]^ Moreover, their immunoregulatory function becomes aberrant, contributing to the immune evasion of AML cells.^[24]^ Notably, AML MSCs always acquire abnormal states, such as inflammatory and senescent phenotypes, accompanied by gene expression profiles significantly distinct from those of normal MSCs, including genes associated with cell cycle regulation, the deoxyribonucleic acid (DNA) damage response, metabolic processes, and immune modulation, collectively contributing to AML progression.^[25, 26, 27]^ Based on these findings, investigating the aforementioned functional and phenotypic changes in MSCs is crucial for evaluating whether these cells are remodeled after CR.

In this study, BM MSCs isolated from patients with de novo AML, patients with AML who achieved complete remission (AML-CR) and healthy controls (HCs) were utilized to evaluate functional changes, including BM MSC differentiation potential, cell apoptosis, cytokine expression and regulatory effects on other cell types. An AML-CR mouse model was established to clarify the functional changes in BM MSCs after CR *in vivo*. Moreover, we investigated the transcriptomic profiles of AML MSCs, CR MSCs and HC MSCs, identified the characteristics of these three groups and speculated that the quantity of senescent BM MSCs might be related to the BM MSC phenotype. Based on these findings, we further explored the MSC senescence state in BM. Our aim was to clarify whether BM MSCs are functionally remodeled after CR and to explore the related alterations in their cellular states, which may provide a new perspective for identifying AML-supporting BM MSCs and improving therapeutic regimens for patients with AML.

## Materials and methods

### Patients and HC samples

Patients with de novo AML, patients with AML-CR at Peking University People’s Hospital were recruited for the study. As Supplementary Table S1-S3 shown, 15 de novo AML patients, 15 AML-CR patients, and 15 HCs were collected in the study. Statistical analysis revealed no significant difference between AML and AML-CR groups in terms of age, sex, and disease subtype. CR was defined as BM blasts < 5%, a hemogram absolute neutrophil count ≥ 1.0 × 10^9^/L, a platelet count ≥ 100 × 10^9^/L, hemoglobin ≥ 100g/L, and the absence of extramedullary disease.^[28]^ AML-CR patient samples were obtained from AML patients who were in CR stably after 5-6 cycles of chemotherapy. Age-matched HC samples (age range, 25-61 years; median age, 42 years) were obtained from healthy donors for allogeneic HSC transplantation.

The study was approved by the Ethics Committee of Peking University People’s Hospital, and written informed consent was obtained from all the subjects, including patients with de novo AML, patients with CR and HCs, in accordance with the Declaration of Helsinki.

### Isolation and culture of BM MSCs

The isolation and culture of BM MSCs followed published protocols.^[29, 30, 31]^ Briefly, BM mononuclear cells (BMMNCs) were isolated through density gradient centrifugation using Ficoll (TBDscience, Tianjin, China). Next, 1 − 3 × 10^7^ BMMNCs per well (typically a 6-well plate) were plated in Dulbecco’s modified Eagle medium (DMEM) with low glucose (Gibco, MA, USA) supplemented with 10% fetal bovine serum (Gibco), 1% penicillin-streptomycin solution (Gibco) and 10 ng/mL FGF-basic (Peprotech, MA, USA), and the cells were further cultured in a humidified incubator with 5% CO_2_ for 72 hours. The medium was then changed every 3-4 days. The representative BM MSC phenotype was identified through flow cytometry as CD34-PE^+^, CD45-PE^+^, CD11b-CD45^-^, CD73-APC^+^, CD90-FITC^+^, CD105-BV421^+^. Detailed information on the antibodies used in this study is provided in Supplementary Table S4.

### BM MSC differentiation assay

For osteogenic differentiation, BM MSCs were cultured in osteogenic medium (Dulbecco’s modified eagle medium [DMEM] -low glucose medium supplemented with 10% fetal bovine serum (FBS), 1% penicillin-streptomycin solution, 0.1 μmol/L dexamethasone (Selleck, TX, USA), 50 μmol/L ascorbic acid (Selleck) and 10 mmol/L β-glycerophosphate (Selleck) for 14 days. An Alizarin Red S Staining Kit for Osteogenesis (Beyotime, Shanghai, China) was used to detect osteogenic differentiation. Specific marker genes, including *IBSP* and *SPP1*, were analyzed on Day 14 after induced differentiation. The primers used for these two genes are shown in Supplementary Table S4.

For adipogenic differentiation, BM MSCs were cultured in adipogenic medium (DMEM-low glucose medium supplemented with 10% FBS, 1% penicillin-streptomycin solution, 1 μmol/L dexamethasone, 500 μmol/L 3-isobutyl-1-methylxanthine (Selleck), and 200 μmol/L indomethacin (Selleck) for 14 days. A Modified Oil Red O Staining Kit (Beyotime) was used to detect osteogenic differentiation. Specific marker genes, including *PLIN2* and *PPARG*, were analyzed on Day 14 after the induction of differentiation. The primers used for these two genes are listed in Supplementary Table S4.

### Flow cytometry analysis

Apoptotic cells were quantified using an APC Annexin V Apoptosis Detection Kit with 7-aminoactinomycin D (AAD) (BioLegend, CA, USA).

For the detection of CD3^+^ cell differentiation, nonadherent cells were collected, and the cell types were determined based on CCR4, CCR6, CD25 and FOXP3 presentation as previously described.^[32]^ For senescence-associated β-galactosidase (SA-β-gal)_-_detection, the cells were collected and stained with a CellEvent senescence green flow cytometry assay kit (Invitrogen, MA, USA) according to the manufacturer’s instructions. The fluorescence intensity was detected using a BD LSRFortessa (Becton Dickinson) and analyzed with FlowJo V10.8.1 software.

### Isolation of BM CD34^+^ cells or CD3^+^ cells and coculture with BM MSCs

BM MSCs were trypsinized, reseeded into 24-well plates and cultured for another 24 hours. BM CD34^+^ cells, AML-derived CD34^+^ cells or CD3^+^ cells were isolated from HC- and AML-BMMNCs using a CD34 (Miltenyi Biotec, Cologne, Germany) or CD3 (Miltenyi Biotec) microbead kit. Then, the cells were resuspended to a final concentration of 1 × 10^5^ CD34^+^ cells/mL with stem span medium (Stem Cell Technology, BC, Canada) containing 100 ng/mL stem cell factor (Peprotech), 100 ng/mL thrombopoietin (Peprotech) and 50 ng/mL FLT-3 ligand (Peprotech) and were directly cocultured for another 5 days.^[9,33,34]^ For CD3^+^ cells, direct-contact coculture was performed with 1 × 10^5^ cells/mL in RPMI 1640 medium (Gibco) supplemented with 10% FBS for 3 days.^[32]^

### Colony-forming unit (CFU) assay

As previously reported,^[35]^ after 5 days of coculture, 4 × 10^3^ CD34^+^ cells were transferred to MethoCult^TM^ H4434 Classic (STEMCELL Technologies Inc., Canada) in 24-well plates and cultured for another 14 days. Colony-forming unit erythroid (CFU-E), burst-forming unit erythroid (BFU-E), colony-forming unit granulocyte/macrophage (CFU-GM), and colony-forming unit granulocyte, erythroid, macrophage and megakaryocyte (CFU-GEMM) measurements were performed using an Olympus IX73 microscope (Olympus, Tokyo, Japan).

### Establishment of the AML-CR mouse model

An AML mouse model was established using 6-8-week-old female BALB/c mice. The AML1-ETO-GFP^+^ AML cells used in our study were derived from a previously established model in which a hybrid C-KIT (HyC-KIT) was engineered by fusing the extracellular and transmembrane domains of murine c-Kit in-frame to the intracellular signaling domain of human C-KIT.^[36]^ Co-expression of AML1-ETO and HyC-KIT (N822K) in hematopoietic progenitor cells resulted in the development of aggressive and fatal AML, faithfully recapitulating key features of human AML pathogenesis.

For the establishment of the AML-CR mouse model, 5 × 10^3^ viable AML1-ETO-GFP^+^ cells were injected into the mice via the tail vein (D0). The AML mouse model was successfully established when the percentage of AML1-ETO-GFP^+^ cells in the peripheral blood exceeded 5%. Then, AML mice received cytarabine (Ara-C) (100 mg/kg) and doxorubicin (DOX) (3 mg/kg) for three consecutive days, followed by Ara-C (100 mg/kg) alone for two additional days. The AML burden was monitored through GFP^+^ cell ratio every two days from D7 regularly, and AML group were sacrificed when GFP^+^ cells in the peripheral blood reached 20%, with age-matched healthy mice used as controls. For the AML-CR group, mice were sacrificed after the proportion of GFP^+^ cells in peripheral blood dropped below 0.5%.^[9,33]^

For flow cytometry detection, the antibodies including CD45-APC, Ter119-APC, CD31-PE-CY7, PDGFRα-BV421, SCA1-BV605, CD51-PE, CD90-APC-CY7, 7AAD were used. The MSCs (CD45^-^Ter119^-^CD31^-^PDGFRα^+^ or CD51^+^), PDGFRα^+^SCA1^+^ (PaS^+^) MSCs (CD45^-^Ter119^-^CD31^-^PDGFRα^+^SCA1^+^), osteoblastic cells (CD45^-^Ter119^-^CD31^-^PDGFRα^+^SCA1^+^), osteoblast progenitor cells (CD45^-^Ter119^-^CD31^-^CD51^+^CD90.2^+^) were analyzed as previously reported.^[29,37]^ Detailed information of the antibodies used in this study is shown in Supplementary Table S4.

### Immunocytochemistry analysis

AML MSCs, CR MSCs and HC MSCs were reseeded onto coverslips (CITOTEST, Jiangsu, China) in 24-well plates. Twenty-four hours later, the cells were fixed with 4% paraformaldehyde (Beyotime) for 15 minutes and blocked with 2.5% BSA (Sigma, MO, USA) containing 0.5% Triton X-100 (Diamond). The cells were incubated with the primary antibody at 4°C overnight and then incubated with the secondary antibody for 1 hour. Then, the cells were mounted on slides with DAPI (Sigma). Fluorescence images were acquired using an Olympus IX73 microscope (Olympus). Detailed information on the primary and secondary antibodies used in this study are shown in Supplementary Table S4.

### Senescence associated β-galactosidase assay

BM MSCs (2 × 10^4^) were trypsinized and reseeded into 12-well plates. A Senescence β-Galactosidase Staining Kit (Beyotime) was used to detect SA-β-gal in BM MSCs according to the manufacturer’s instructions. Briefly, cells were fixed at room temperature for 15 minutes, followed by incubation with the staining solution prepared according to the manufacturer’s instructions. The samples were then incubated overnight at 37 °C, and the cells dyed blue were analyzed.

### Ribonucleic acid sequencing (RNA-seq) and bioinformatic analysis

RNA-seq was performed on BM MSCs from patients with AML, patients with CR and HCs. Paired-end clean reads were aligned to GRCH38 using HISAT2 (Version 2.0.5), and FeatureCounts (Version 1.5.0-p3) was used to count the number of reads. Differentially expressed genes were identified using DESeq2 (version 1.20.0). Genes with the criteria of |log2 (fold change)| > 1 & *P* value < 0.05 were considered high-confidence differentially expressed genes. Gene Ontology (GO) enrichment analysis of the differentially expressed genes was executed using the clusterProfiler R package (version 3.8.1). Gene set enrichment analysis (GSEA) was performed using MSigDB (version 5.1), and the gene set used in the GSEA was "C5. GO. BP".

### Statistical analysis

Statistical analysis was performed using Prism 8.0 (Graph Pad Software Inc., CA, USA). The experiments were performed at least in triplicate, and the data are presented as the means *±* standard errors of the means (SEMs). For comparisons among the three groups (AML, CR, and HC), one-way analysis of variance (ANOVA) was applied, followed by Tukey’s multiple comparison test to determine pairwise differences. Other statistical significance was evaluated by Student’s *t* test. Differences were considered statistically significant when the *P* value was < 0.05 (*P < 0.05, **P < 0.01 and ***P < 0.001).

## Results

### The functions of BM MSCs were modified when patients with AML achieved CR

**Figure 1:**
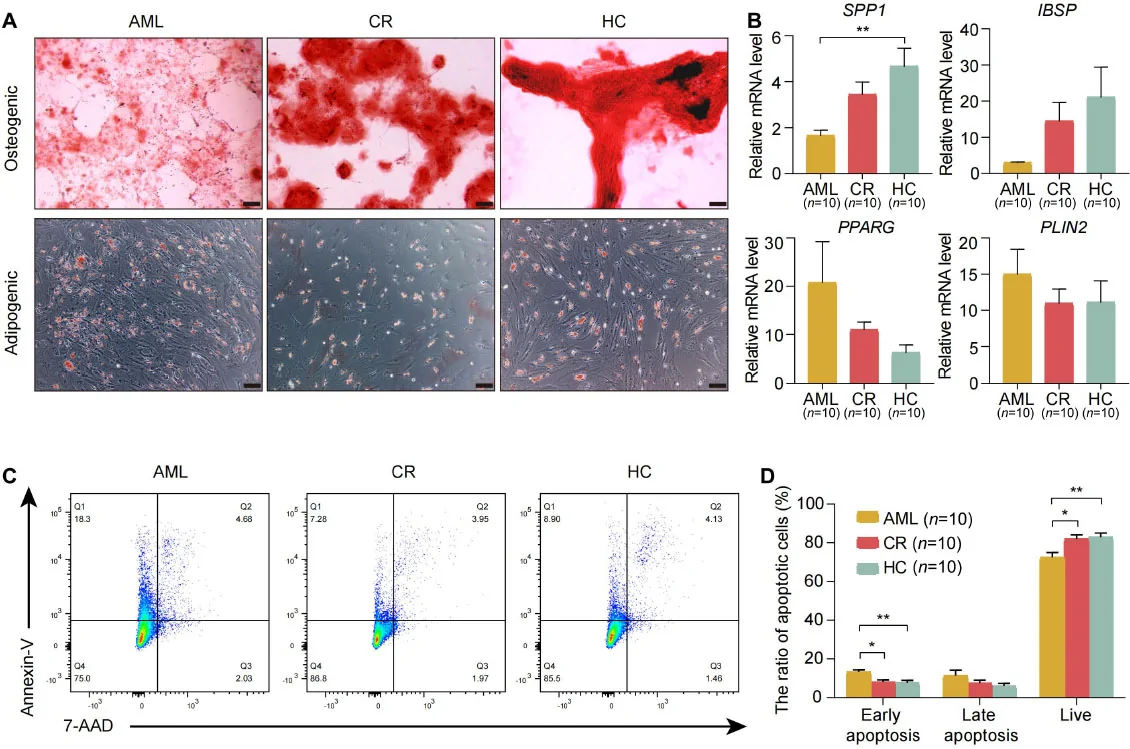
The evaluation of functional changes of AML MSCs, CR MSCs and HC MSCs. (A) Representative images of alizarin red S staining for osteogenesis (upper panel) or oil red O staining for adipogenesis (lower panel) in AML MSCs, CR MSCs and HC MSCs. Each MSC group contains 10 samples (*n* = 10). Scale bar represents 100 μm. (B) qRT-PCR analysis showing the expression of osteogenic marker genes (*IBSP*, *SPP1*) and adipogenic marker genes (*PLIN2*, *PPARG*). (C, D) Flow cytometry analysis to determine the apoptotic MSCs among patients with AML, patients with CR and HC. Each MSC group contains 10 samples (*n* = 10). Statistical analyze is shown in (D). *P* values were calculated using one-way ANOVA, and *P* < 0.05 was considered statistically significant. *P < 0.05, **P < 0.01. AML: acute myeloid leukemia; MSCs: mesenchymal stem cells; CR: complete remission; HC: healthy control; qRT-PCR: quantitative real-time reverse transcription PCR.

To evaluate BM MSC function, the differentiation potential of osteogenic and adipogenic cells and the apoptotic cell ratio were compared among patients with de novo AML (AML MSCs), patients with CR (CR MSCs) and healthy controls (HC MSCs). As shown in Figure 1A, compared with that of HC MSCs, the osteogenic capacity of AML MSCs was lower, whereas the adipogenic capacity was greater, which was consistent with published articles.^[38,39]^ The decreased expression levels of osteogenesis-related genes (*IBSP* and *SPP1*) and increased expression levels of adipogenesis-related genes (*PLIN2* and *PPARG*) further confirmed the above results (Figure 1B). Moreover, the ratio of apoptotic AML MSCs was greater than that of HC MSCs (Figure 1C, 1D). Interestingly, compared with that of AML MSCs, the osteogenic capacity of CR MSCs was also greater, the adipogenic capacity was lower (Figure 1A), and the expression levels of osteogenesis- and adipogenesis-related genes were similar to those of HC MSCs compared with those of AML MSCs (Figure 1B). Additionally, the ratio of apoptotic CR MSCs was also lower than that of AML MSCs (Figure 1C, 1D). Taken together, these results suggest that the functions of BM MSCs are modified after CR and that these functions are more similar to those of HC MSCs.

### The regulatory effects of BM MSCs on CD34^+^ cells or CD3^+^ T cells after CR were improved such that they became more similar to those of HC MSCs

**Figure 2:**
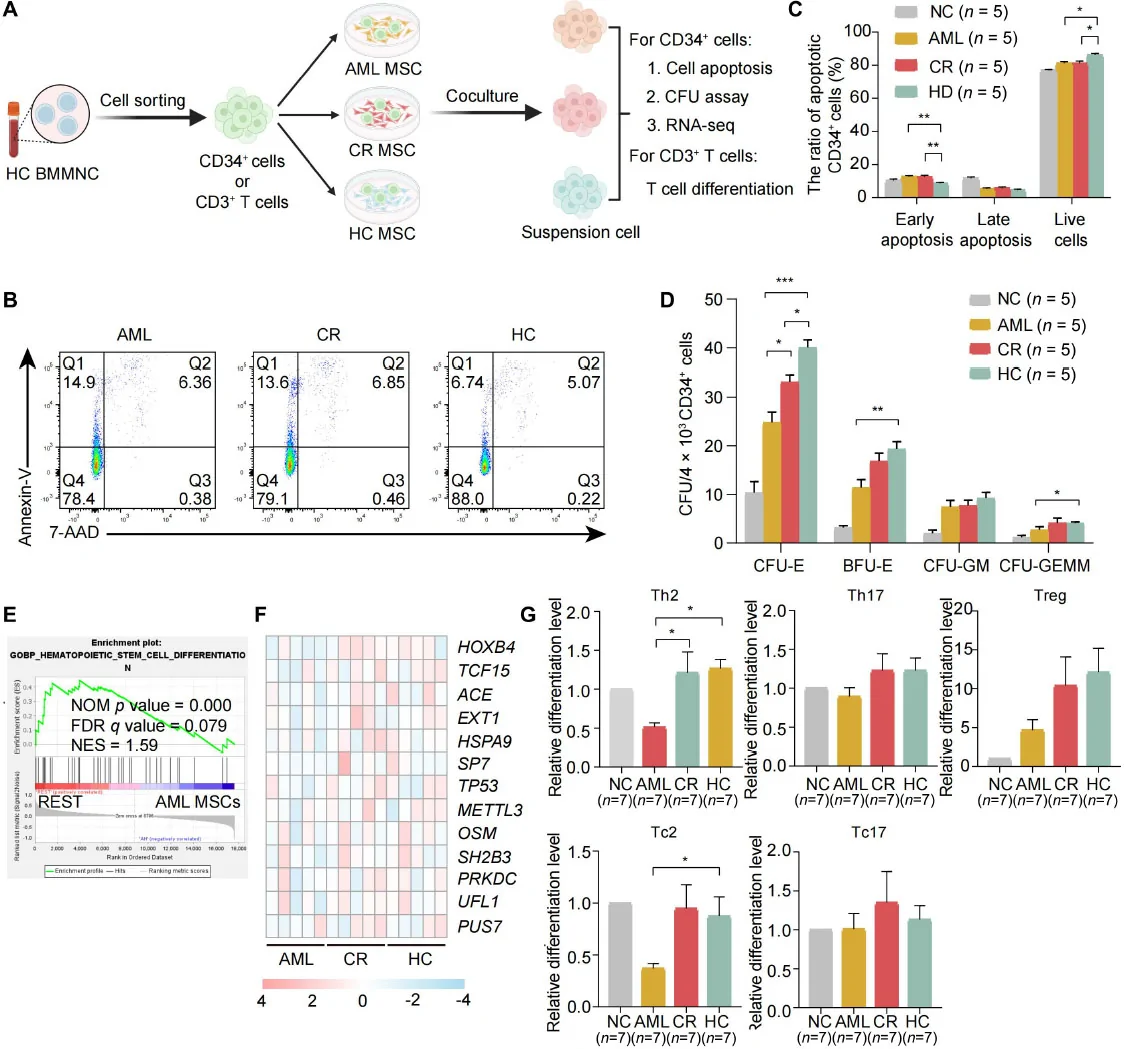
The detection of regulatory role of BM MSCs on CD34^+^ and CD3^+^ cells. (A) Schematic diagram of the experimental design for the BM MSC coculture process with CD34^+^ and CD3^+^ cells isolated from HC BMMNCs. (B, C) Flow cytometry analysis to determine the apoptotic CD34^+^ cells after coculture with AML MSCs, CR MSCs and HC MSCs. Each MSC group contains 5 samples (*n* = 5). Statistical analysis is shown in (C). (D) The CFU assay to determine the plating efficiency of CD34^+^ cells coculture with AML MSCs, CR MSCs and HC MSCs, including CFU-E, BFU-E, CFU-GM and CFU-GEMM. Each MSC group contains 5 samples (*n* = 5). (E) GSEA analysis of the indicated genes in AML MSCs or REST (CR MSCs and HC MSCs). (F) Heatmap of the hematopoietic stem cell differentiation related genes of CD34^+^ cells after coculture with AML MSCs, CR MSCs and HC MSCs. Each MSC group contains 5 samples (*n* = 5). (G) Statistical analysis to determine the Th2 cells, Th17 cells, Treg cells, Tc2 cells and Tc17 cells. Each MSC group contains 7 samples (*n* = 7). *P* values were calculated using one-way ANOVA, and *P* < 0.05 was considered statistically significant. *P < 0.05, **P < 0.01, ***P < 0.001. BM: bone marrow; MSCs: mesenchymal stem cells; BMMNCs: BM mononuclear cells; AML: acute myeloid leukemia; CR: complete remission; HC: healthy control; CFU-E: colony-forming unit erythroid; BFU-E: burst-forming unit erythroid; CFU-GM: colony-forming unit granulocyte/macrophage; CFU-GEMM: colony-forming unit granulocyte, erythroid, macrophage and megakaryocyte.

To compare the regulatory roles of BM MSCs in supporting normal HSCs and immunoregulation, CD34^+^ cells or CD3^+^ T cells sorted from HC were cocultured with AML MSCs, CR MSCs or HC MSCs separately (Figure 2A). Compared with those of HC MSCs, the ratio of live CD34^+^ cells cocultured with AML MSCs was lower, and the number of early apoptotic CD34^+^ cells was greater (Figure 2B, 2C). In addition, the efficiencies of CD34^+^ cells cocultured with AML MSCs in the formation of CFU-E, BFU-E and CFU-GEMM were lower (Figure 2D). Furthermore, although live and early apoptotic CD34^+^ cells showed the same tendency as AML MSCs when cocultured with CR MSCs (Figure 2B, 2C), the efficiencies of CD34^+^ cells in the formation of CFU-E, BFU-E and CFU-GEMM were greater than those of AML MSCs (Figure 2D). In addition, GSEA and heatmap analysis revealed that the expression levels of HSC differentiation-related genes were also positively correlated with CR MSCs and HC MSCs (Figure 2E, 2F). These results indicate that the ability of CR MSCs to regulate CD34^+^ cells is similar to that of HC MSCs.

To investigate the immunoregulatory effects of BM MSCs, CD3^+^ T cells were detected through flow cytometry after a coculture assay. The gating strategy for T-cell detection is shown in Supplementary Figure S1A. Compared with AML MSCs, CD3^+^ T cells cocultured with CR MSCs rescued the immunosuppression of AML cells against BM MSCs, as the ratios of Th cells and Tc cells, including Th2 and Tc2 cells, significantly increased, and the proportions of Treg, Th17 and Tc17 cells slightly increased (Figure 2G, Supplementary Figure S1B). The changes in CD3^+^ T cells were also consistent with those in HC MSCs compared with those in AML MSCs. These data indicate that CR MSCs promote the differentiation of CD3^+^ T cells into functional immune cells. Combined with the regulatory effect of BM MSCs on CD34^+^ and CD3^+^ cells, these results suggest that the regulatory role of CR MSCs in supporting normal HSCs and immunoregulation is remodeled such that they are more similar to those of HC MSCs.

### The expression levels of cytokine-related genes involved in the immune response and hematopoietic regulation were upregulated after CR

**Figure 3:**
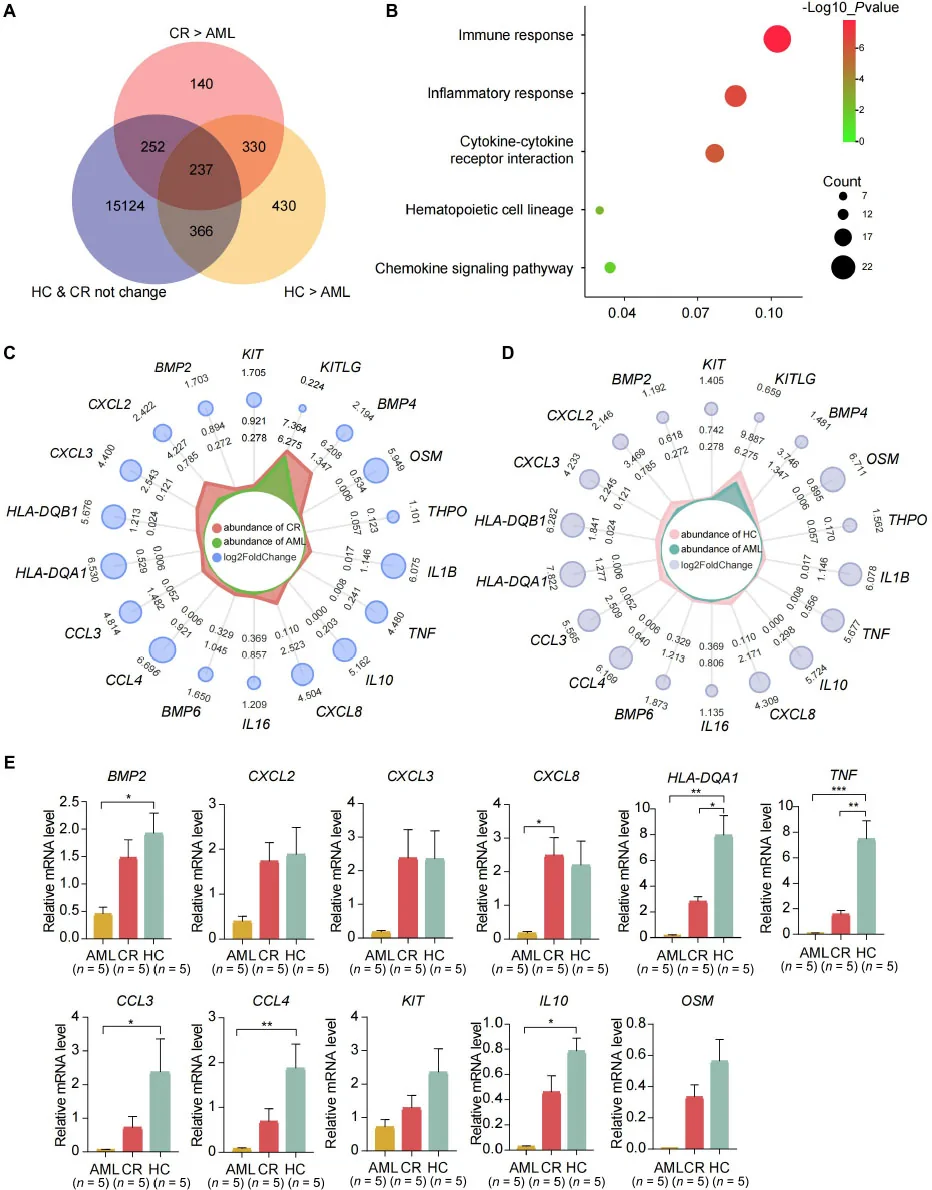
The detection of cytokine related gene expression among AML MSCs, CR MSCs and HC MSCs. (A) Venn diagrams of the up-regulated genes in CR MSCs and HC MSCs compared to AML MSCs, whereas no change between MSCs from patients with CR and HC. (B) GO enrichment analysis of overlap genes in Figure 3A. (C, D) The differential expressed cytokine related genes in CR MSCs (C) or HC MSCs (D) when compared with AML MSCs. (E) qRT-PCR to verify the expression of differential cytokine related genes. Each MSC group contains 5 samples (*n* = 5). *P* values were calculated using one-way ANOVA, and *P* < 0.05 was considered statistically significant. *P < 0.05, **P < 0.01, ***P < 0.001. AML: acute myeloid leukemia; MSCs: mesenchymal stem cells; CR: complete remission; HC: healthy control; qRT-PCR: quantitative real-time reverse transcription PCR.

Because the regulatory function of BM MSCs is restored after CR and BM MSCs play a regulatory role by secreting cytokines, we next proceeded to examine the expression levels of cytokine-related genes in BM MSCs. We identified 237 genes that were upregulated in both CR MSCs and HC MSCs compared with AML MSCs (Figure 3A). GO analysis of these 237 genes revealed that the differentially expressed genes were significantly enriched in the immune response, hematopoietic cell lineage, and cytokine-cytokine receptor interactions (Figure 3B). Cytokines with reported functions in immunity and hematopoiesis were selected from the 237 up-regulated genes for validation. After radar image construction and verification at the RNA level, we confirmed that key cytokines related to the immune response and hematopoiesis in CR MSCs and HC MSCs were upregulated compared with those in AML MSCs, including *BMP2*, *CXCL2*, *CXCL3*, *CXCL8*, *HLA-DQA1* and *TNF*; the expression levels of *CCL3*, *CCL4*, *KIT*, *IL10*, and *OSM* also tended to be upregulated in CR MSCs compared with AML MSCs (Figure 3C-3E, Supplementary Figure S2). These findings indicate that the upregulated expression of these cytokines contributes to the recovery of BM MSC regulatory functions.

### The functions of MSCs were restored when CR was achieved in the AML-CR mouse model

**Figure 4:**
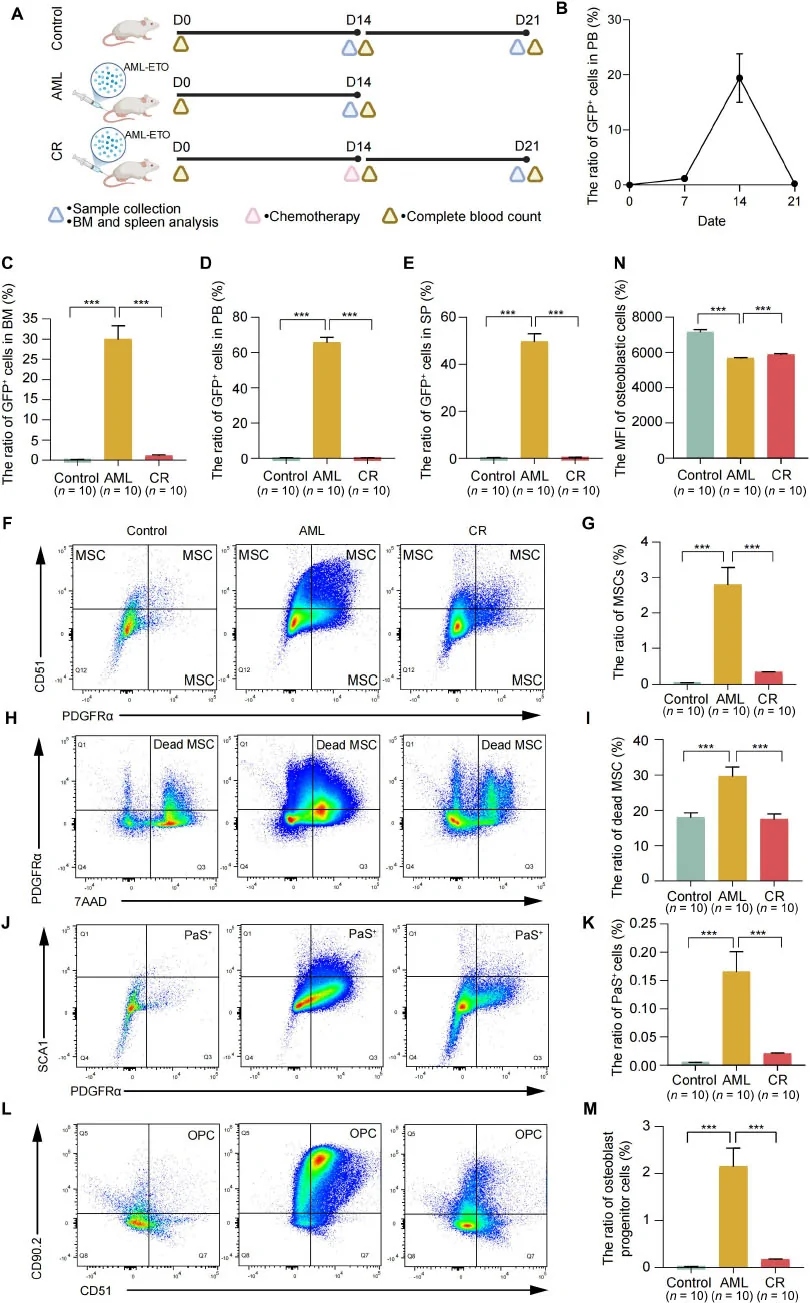
The evaluation of frequency and functions of BM MSCs through AML-CR mouse model. (A) Schematic diagram of the experimental design for establishment of AML-CR mouse model. (B) Flow cytometry analysis to determine the dynamical ratio of GFP^+^ cells in peripheral blood. (C-E) Flow cytometry analysis to determine the ratio of GFP^+^ cells in bone marrow (C), peripheral blood (D) and spleen (E) when sample collection. (F-M) Flow cytometry analysis to determine the ratio of total BM MSCs (F), dead MSCs (H), PaS^+^ MSCs (J) and osteoblastic progenitor cells (L). Statistical analyze are shown in G, I, K, M separately. N. MFI analysis to evaluate the frequency of osteoblastic cells. The AML, CR, Control group consists of 10 mice separately (n = 10). *P* values were calculated using one-way ANOVA, and *P* < 0.05 was considered statistically significant. ***P < 0.001. BM: bone marrow; MSCs: smesenchymal stem cells; AML-CR: acute myeloid leukemia who achieved complete remission; GFP: green fluorescent protein; PB: peripheral blood; SP: spleen; OPC: osteoblastic progenitor cells.

To further investigate functional remodeling after CR, we detected the quantity and functions of BM MSCs in an AML-ETO-induced AML mouse model. AML mice were treated with Ara-C and DOX in combination to achieve CR (Figure 4A). We dynamically monitored the proportion of GFP^+^ cells in the peripheral blood and performed routine blood examination to assess the establishment of a mouse model (Figure 4B, Supplementary Figure S3A-S3D). As shown in Figure 4C-4E and Supplementary Figure S3E, the disease status was determined by analyzing the GFP^+^ cell content in peripheral blood and BM at the time of sample collection, while the extent of AML cell infiltration was evaluated based on the proportion of GFP^+^ cells in the spleen.

Following the successful establishment of the AML-CR mouse model, flow cytometry was used to determine the MSC conditions (Supplementary Figure S3F). Compared with that of control mice, the ratio of AML MSCs was significantly increased, as identified by the expression levels of PDGFRα and CD51, whereas it was further decreased after CR (Figure 4F, 4G). Consistent with the results of the *in vitro* MSC apoptosis assay, the death of AML MSCs was greater than that of the control, and the ratio of apoptotic CR MSCs was lower than that of AML MSCs (Figure 4H, 4I). Interestingly, the PDGFRα^+^SCA1^+^ (PaS^+^) MSCs re-emerges in AML and subsequently decreases after CR (Figure 4J, 4K), which subtype were reported that emerges during embryonic development but absent in mice over 2 weeks old and play important roles in HSC behavior;^[37]^ suggesting the presence of a functionally abnormal MSC subset in AML that partially recovers after CR. Furthermore, Consistent with previous work,^[40,41]^ we also observed a significant increase in osteoblast progenitors and a reduction in osteoblastic cells in the AML group compared with controls, whereas in the CR group, the proportion of osteoblast progenitors returned to normal and the number of osteoblastic cells increased compared to the AML group, indicating the differentiation potential of BM-MSCs was recovered after CR (Figure 4L-4N). Taken together, the above results suggest that BM MSC homeostasis is disrupted in patients with AML but further recovers after CR through chemotherapy.

### The transcription profile and characteristics of CR MSCs were remodeled and transformed to be more similar to those of HC MSCs

**Figure 5:**
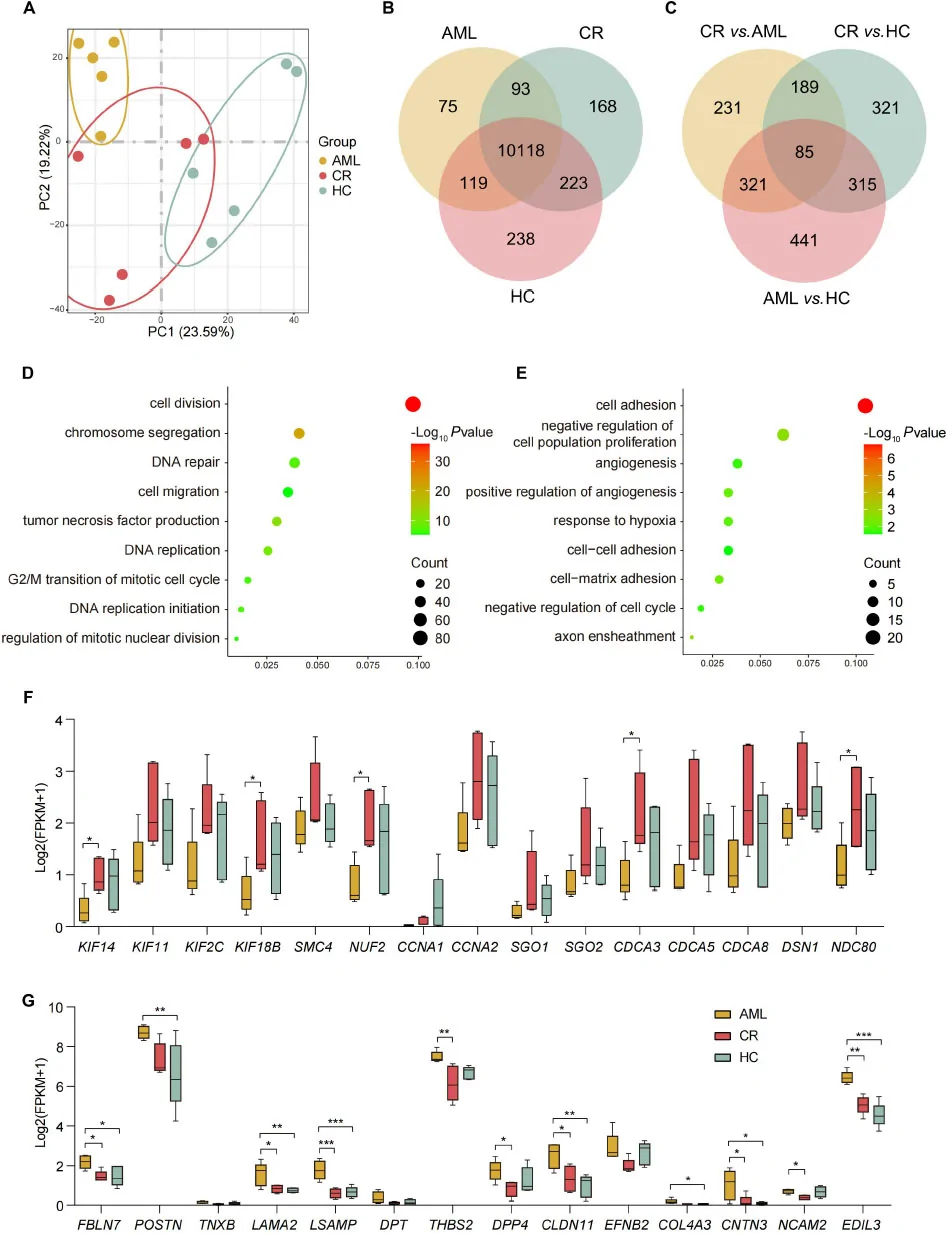
Transcriptome-wide RNA expression analysis of AML MSCs, CR MSCs and HC MSCs. (A) PCA analysis of AML MSCs, CR MSCs and HC MSCs. The x-axis represents the first principal component (PC1), and the y-axis explains the second largest variance (PC2). (B, C) Venn diagrams of the expressed (B) or differential expressed (C) genes among AML MSCs, CR MSCs and HC MSCs. (D) GO analysis of the up-regulated genes in CR MSCs compared to AML MSCs. (E) GO analysis of the down-regulated genes in CR MSCs compared to AML MSCs. (F, G) The RNA expression of genes related to cell division (F) and cell adhesion (G). *P* values were calculated using one-way ANOVA, and *P* < 0.05 was considered statistically significant. *P < 0.05, **P < 0.01, ***P < 0.001. RNA: ribonucleic acid sequencing; AML: acute myeloid leukemia; MSCs: mesenchymal stem cells; CR: complete remission; HC: healthy control; PCA: principal component analysis.

To further investigate the potential mechanisms underlying the restoration of BM MSC function after CR, we compared the transcription profiles of AML MSCs, CR MSCs and HC MSCs isolated from patients and HC. Principal component analysis (PCA) revealed that AML MSCs were markedly different from HC MSCs, and CR MSCs were in an “intermediate state” (Figure 5A). CR MSCs still retain certain AML-associated residual features, manifested as compared with HC MSCs, 121 upregulated genes were identified both in AML MSCs and CR MSCs, which were functionally enriched in “cell migration”, “TGF-β receptor signaling” and “cell-cell junction organization” (Supplementary Figure S4A). In addition, we also identified 379 downregulated genes, which were mainly enriched in “Chemotaxis”, “Cell apoptosis”, “cell differentiation” (Supplementary Figure S4B). Interestingly, a subset of growth- and niche-supporting senescence-associated secretory phenotype (SASP) factors, including VEGFA, FGF2, IGFBP3, MMP2, and GDF15, displayed a progressive decrease across AML, CR and HC samples (Supplementary Figure S4C). These genes are well-known mediators of angiogenesis, extracellular matrix remodeling and hematopoietic support.^[42, 43, 44, 45, 46]^ The elevated expression of these factors in AML MSCs suggested that leukemic cells may reprogram the stromal niche toward a trophic and angiogenic phenotype favorable for leukemia progression. Importantly, the level of SASP factors in CR MSCs still remained higher than those in HC MSCs, indicating that CR MSCs retain residual leukemia-associated features that may contribute to impaired hematopoietic recovery.

To further determine the transcription profile remodeling of CR MSCs, a greater number of coexpressed genes were detected in CR MSCs and HC MSCs (*n* = 223); and genes that were differentially expressed between AML MSCs and HC MSCs (*n* = 441) also supported this finding (Figure 5B, 5C). Compared with those in AML MSCs, upregulated genes in CR MSCs were enriched in processes such as cell division, DNA repair, and DNA replication; downregulated genes were significantly enriched in cell adhesion (Figure 5D, 5E). In addition, compared with those in AML MSCs, genes related to cell division and DNA replication were upregulated, whereas genes related to cell adhesion were downregulated, and the variation tendency of these genes was similar to that in HC MSCs (Figure 5F, 5G). The above results suggest that the transcription profile of CR MSCs is transformed to that of HC MSCs and that CR MSCs are more similar to HC MSCs. Because cell cycle arrest and DNA replication disorders are important phenotypes of cell senescence, combined with the functional changes in BM MSCs, we speculated that the reduction in the number of senescent BM MSCs might be the reason for the functional improvement in CR MSCs.

### The quantity of senescent cells in CR MSCs and HC MSCs was lower than that in AML MSCs

**Figure 6:**
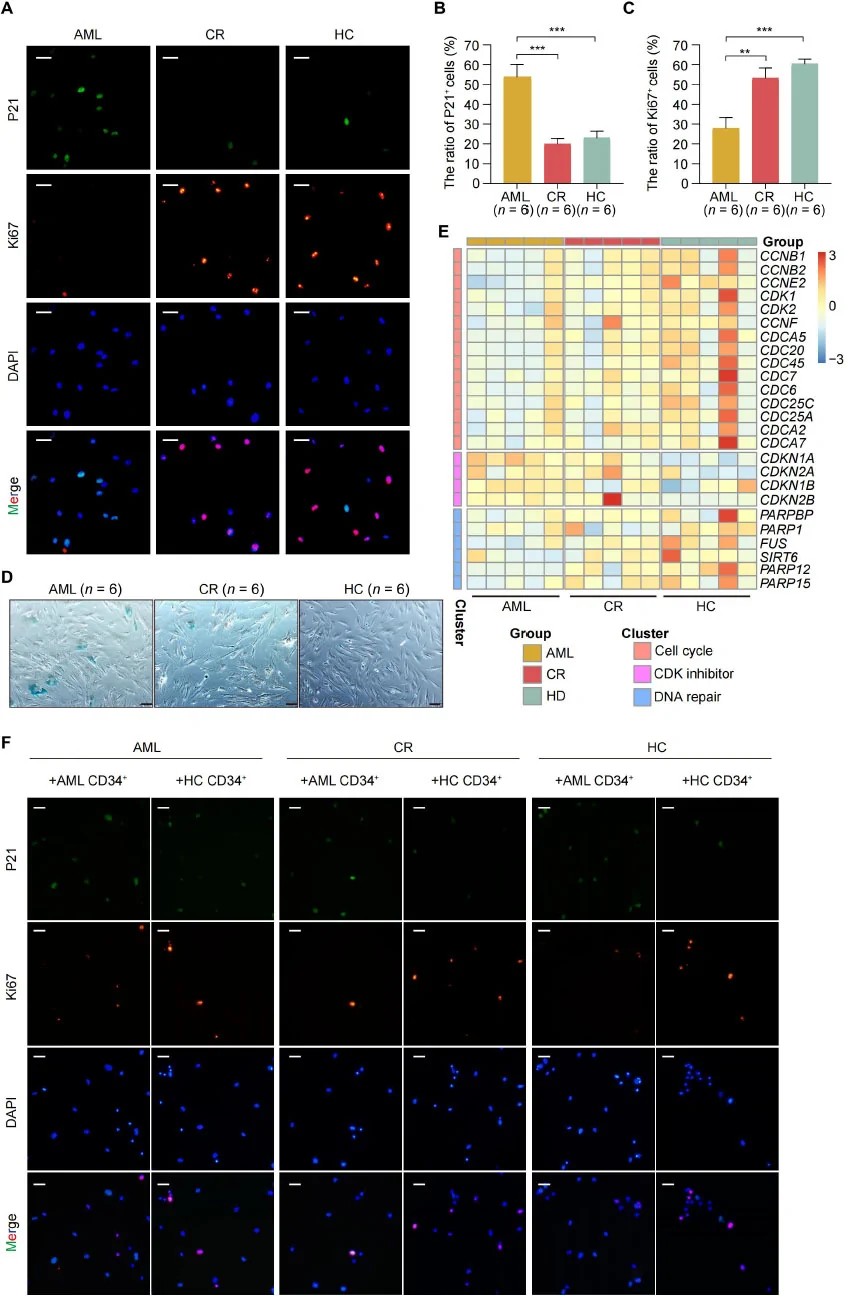
The detection of senescent MSCs in patients with AML, patients with CR and HC. (A) Representative images of immunofluorescence staining of P21 and Ki67 in AML MSCs, CR MSCs and HC MSCs, respectively. Each MSC group contains 6 samples (*n* = 6). Scale bar represents 50 μm. (B, C) Statistical analysis of P21^+^ (B) and corresponding Ki67^+^ (C) MSCs in these three groups. (D) Representative images of SA-β-gal staining in AML MSCs, CR MSCs and HC MSCs. Each MSC group contains 6 samples (*n* = 6). Scale bar represents 100 μm. (E) The RNA expression of cell senescence related genes, including CDK inhibitor, cell cycle and DNA repair. (F) Representative images of immunofluorescence staining of P21 and Ki67 in AML MSCs, CR MSCs and HC MSCs coculture with AML CD34^+^ cells or HC CD34^+^ cells, respectively. Each MSC group contains 6 samples (*n* = 6). Scale bar represents 50 μm. *P* values were calculated using one-way ANOVA, and *P* < 0.05 was considered statistically significant. **P < 0.01, ***P < 0.001. MSCs: mesenchymal stem cells; AML: acute myeloid leukemia; CR: complete remission; HC: healthy control; SA-β-gal: senescence-associated β-galactosidase; RNA: ribonucleic acid sequencing; CDK: cyclin-dependent kinase; DNA: deoxyribonucleic acid.

To assess the cellular senescence status of BM MSCs, we detected the levels of the cellular senescence markers P21, P27, Ki67, and SA-β-gal in AML MSCs, CR MSCs and HC MSCs. Compared with those of AML MSCs, the number of cells positive for the cyclin-dependent kinase (CDK) inhibitors P21 and P27 was lower, and the number of Ki67-positive cells was increased (Figure 6A-6C, Supplementary Figure S5A-S5C), whereas the number of SA-β-gal positive cells was significantly lower in CR MSCs (Figure 6D). HC MSCs also exhibited the same changes, characterized by increased Ki67-positive cells and decreased P21-, P27- and SA-β-gal positive cells (Figure 6A-6D, Supplementary Figure S5A-S5C). Furthermore, RNA-seq analysis revealed the same molecular pattern, characterized by increased expression levels of cell cycle- and DNA repair-related genes and decreased expression levels of CDK inhibitors in CR MSCs and HC MSCs compared with those in AML MSCs (Figure 6E). These findings indicate that alterations in the senescence status of MSCs are associated with the recovery of their functional properties.

### CR MSCs regain the characteristics of reinduced senescence

Previous studies have demonstrated that normal MSCs can be induced into a senescent state by AML cells.^[26,47]^ To further investigate whether CR MSCs regain their normal functional properties, we assessed the senescence status of AML MSCs, CR MSCs and HC MSCs following coculture with either AML-derived CD34^+^ cells or CD34^+^ cells from HC (Supplementary Figure S5D), and the purity consistently exceeded 90% with high cell viability, thereby minimizing the possibility of non-specific results (Supplementary Figure S5E). Consistent with HC MSCs, CR MSCs transformed to senescent cells after coculture with AML CD34^+^ cells, characterized by increased P21-positive cells, decreased Ki67-positive cells (Figure 6F, Supplementary Figure S5F-S5G) and increased SA-β-gal positive cells (Supplementary Figure S5H). However, for AML MSCs, removal of AML cells could not reverse the cellular senescence state. Taken together, these findings suggest that MSCs derived from patients with CR regain the characteristics of reinduced senescence and exhibit characteristics similar to those of MSCs derived from HCs. The lower prevalence of senescent MSCs in patients with CR than in patients with AML may represent a key point underlying the restoration of CR MSC function.

### AML MSCs exhibit a senescence phenotype similar to that of normal MSCs in which senescence is induced

**Figure 7:**
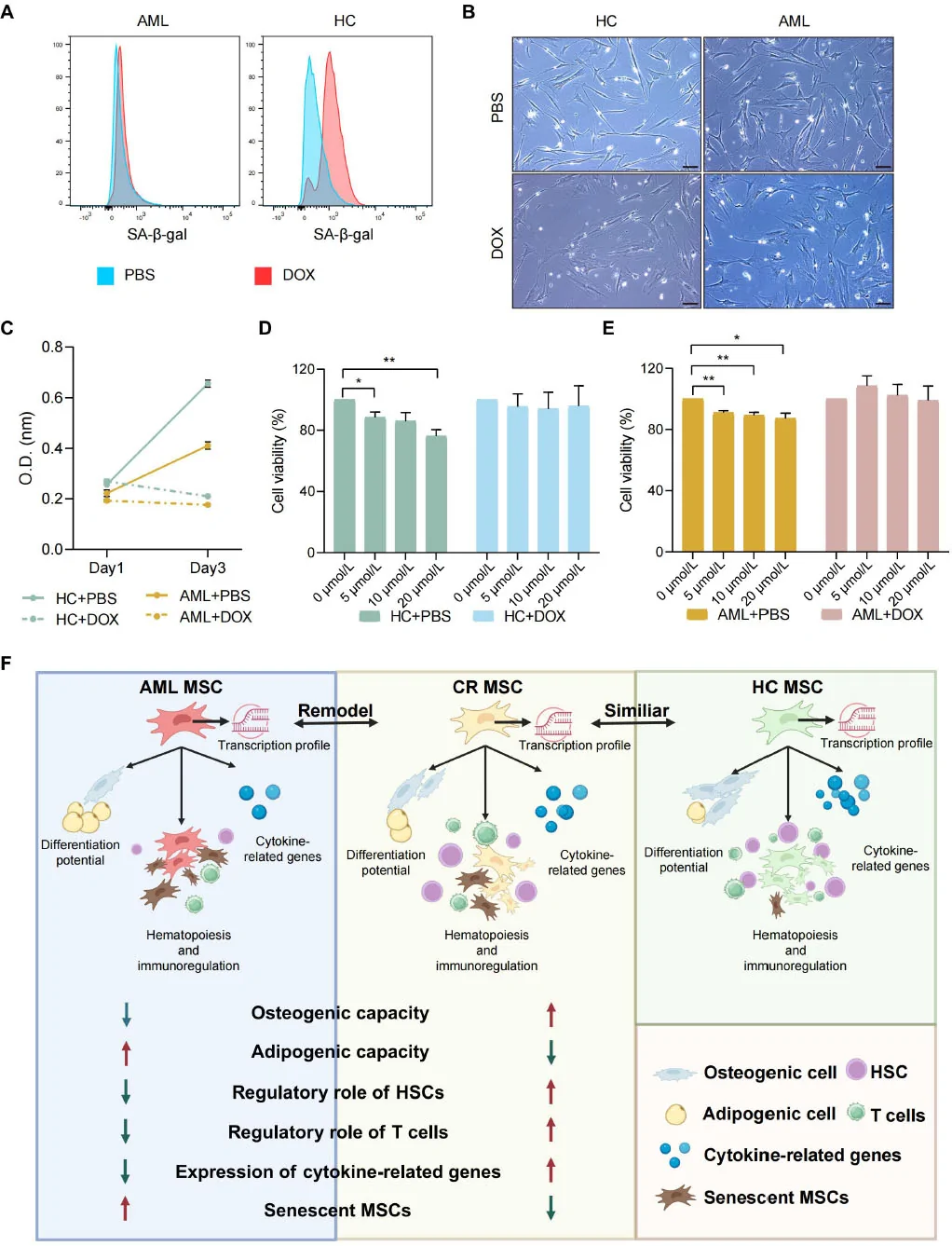
The comparison of cellular state of MSCs in AML patients and HC. (A) Flow cytometry analysis to compare the SA-β-gal^+^ cells treated with DOX and PBS. (B) Representative images of MSC morphology of cells treated with DOX and PBS. Scale bar represents 100 μm. (C) CCK-8 assay to compare the potential of MSC proliferation. (D, E) Cell viability assay showing the chemosensitivity of MSCs treated with DOX or PBS. Representative results from six replicates were shown here. (F) Graphical abstract showing the functionally remodeled of CR MSCs, which were transformed to be more similar with HC MSCs. The graphical abstract was created with BioRender.com, with permission. *P* values were calculated using two-tailed Student’s *t*-test, and *P* < 0.05 was considered statistically significant. *P < 0.05, **P < 0.01. MSCs: mesenchymal stem cells; AML: acute myeloid leukemia; HC: healthy control; SA-β-gal: senescence-associated β-galactosidase; DOX: doxorubicin; PBS: phosphate-buffered saline; CR: complete remission.

To further elucidate the cellular state of MSCs in leukemia, we employed an in vitro senescence induction model to investigate functional changes. Treatment with DOX induced the expression of the senescence marker β-galactosidase in MSCs (Figure 7A). However, the increase in the proportion of senescent cells was less pronounced in AML MSCs than in HC MSCs. Notably, following DOX treatment, AML MSCs exhibited an enlarged and flattened morphology, upregulation of the cell cycle inhibitor P21, downregulation of the proliferation marker Ki67 and cell cycle arrest (Figure 7B, 7C; Supplementary Figure S6A-S6C). These phenotypic and functional changes were largely consistent with those observed in DOX-treated HC MSCs, indicating typical features of cellular senescence. Furthermore, drug sensitivity assays and apoptosis kinetics analysis demonstrated that senescent MSCs exhibited increased resistance to the chemotherapeutic agent Ara-C, suggesting that the reduction in senescent MSCs after CR may not result from direct chemotherapeutic cytotoxicity but rather may involve other regulatory mechanisms (Figure 7D, 7E, Supplementary Figure S7). Collectively, these findings indicate that senescent AML MSCs exhibit characteristics similar to those of normal senescent MSCs, implying that the dysfunction observed in AML MSCs may be attributed to an increased proportion of senescent cells.

## Discussion

The present study demonstrates the functional remodeling of BM MSCs isolated from patients with de novo AML, patients with CR and HCs, and the results were further verified in an AML-CR mouse model. Moreover, the transcriptomic profile of CR MSCs was more similar to that of HC MSCs. Through bioinformatic analysis and senescent BM MSC detection, we demonstrated that the reduction in the number of senescent BM MSCs was associated with the functional remodeling of CR MSCs (Figure 7F).

Previous studies have shown that the function of BM MSCs is to support leukemia cells during AML.^[48]^ The results of the present study demonstrated that AML-modified BM MSCs can be remodeled to support normal hematopoiesis and restore immunoregulatory function after CR. Although the precise mechanisms underlying the remodeling and functional recovery of BM MSCs remain to be elucidated, our data suggest that this process may represent a critical factor in the restoration of normal hematopoiesis in patients with AML.

Previous studies have mainly focused on BM MSC alterations during the AML stage. For example, leukemia cells interact with MSCs, reprogram their transcriptome toward abnormal phenotypes such as senescence, whereas targeting these aberrant MSCs can improve AML progression.^[13,26]^ In contrast, our study systematically investigates BM MSCs during CR, a disease stage that has not been well characterized in prior work. Our findings demonstrate that, CR MSCs undergo functional remodeling toward a state more similar to that of HC MSCs, targeting senescent MSCs after CR and preserving the homeostasis of CR MSCs may therefore better support hematopoietic recovery. Our work complements previous research by shifting the perspective from AML-driven dysfunction to post-treatment functional recovery, with important implications for understanding hematopoietic restoration and improving patient outcomes.

Cellular senescence is a cell state involved in various physiological processes and age-related diseases and is characterized by irreversible cell cycle arrest and widespread changes in chromatin organization and gene expression.^[27,49,50]^ Normal hematopoiesis has been shown to be disrupted by cellular senescence.^[25,26,51]^ The removal of senescent cells from the microenvironment could attenuate the effects of age-related degenerative diseases, such as the regenerative healing model of osteoarthritis.^[52]^ Furthermore, previous work revealed that the number of senescent MSCs in patients with tumors was greater than that in HC.^[53, 54, 55, 56, 57, 58]^ In the present study, we demonstrated that the quantity of senescent cells was decreased in CR MSCs and that their functions were restored, suggesting that the reduction in the number of senescent MSCs might have a positive effect on normal hematopoietic recovery. However, the molecular mechanisms driving the reduction of senescent MSCs after CR remain to be elucidated. One possibility is that non-senescent MSCs outcompete senescent counterparts, leading to their relative decline; in parallel, partial immune reconstitution after CR may contribute to the clearance of senescent cells, potentially through NK cells, macrophages, or cytotoxic T lymphocytes.^[59,60]^ In recent years, several senolytics including dasatinib plus quercetin, fisetin, *etc*. have demonstrated efficacy in eliminating senescent cells in preclinical models.^[61, 62, 63]^ Together, these considerations suggest that both intrinsic MSC dynamics and extrinsic immune- or drug-mediated mechanisms may account for the reduction of senescent MSCs observed after CR.

Nevertheless, further studies required to evaluate whether therapeutic clearance of senescent MSCs could further enhance hematopoietic recovery and therefore improve the prognosis of AML patients. In addition, the relationship between functional recovery of CR MSCs and prognosis of patients still requires further exploration.

## Conclusion

Our results describe the differences in the functions, transcriptomic profiles and cellular senescence states of MSCs from patients with AML, patients with CR and healthy individuals. Our work provides a new perspective indicating that AML-modified BM MSCs can be remodeled to support normal HSCs in patients with CR. Furthermore, targeting aberrant BM MSCs may contribute to AML therapy, promote normal hematopoietic recovery and the prevention of AML relapse.

## Supplementary Information

Supplementary materials are only available at the official site of the journal (www.intern-med.com).

## Supplementary Material

Supplementary Material Details
