# Supplementary material for: Bone marrow mesenchymal stem cells are functionally remodeled after complete remission of acute myeloid leukemia
Source: J Transl Int Med. 2026 Aug 24;14(4):563–78. doi: 10.1515/jtim-2026-0048 (PMC13509149; doi:10.1515/jtim-2026-0048)
Supplement: Supplementary file 1 [file jtim-2026-0048_sm.pdf]

Supplementary Materials

Bone Marrow Mesenchymal Stem Cells Are Functionally Remodeled after Complete Remission of Acute Myeloid Leukemia

Authors: Zhi-Wei Zhang[1], Zhen-Kun Wang[1], Xin-Yan Zhang[1], Dan-Dan Chen[1], Chen-Yuan Li[1], Li-Juan Hu[1], Zheng-Li Xu[1], Xiao-Jun Huang[1,2*], Yuan Kong[1*]

Affiliations:

[1]Peking University People's Hospital, Peking University Institute of Hematology, National Clinical Research Center for Hematologic Disease, Beijing Key Laboratory of Cell and Gene Therapy for Hematologic Malignancies, Peking University, Beijing, China;

[2]Peking-Tsinghua Center for Life Sciences, Academy for Advanced Interdisciplinary Studies, Peking University, Beijing, China.

Correspondence: Yuan Kong[1], Xiao-Jun Huang[1,2]

* Yuan Kong and Xiao-Jun Huang are co-corresponding authors

Supplementary Figures

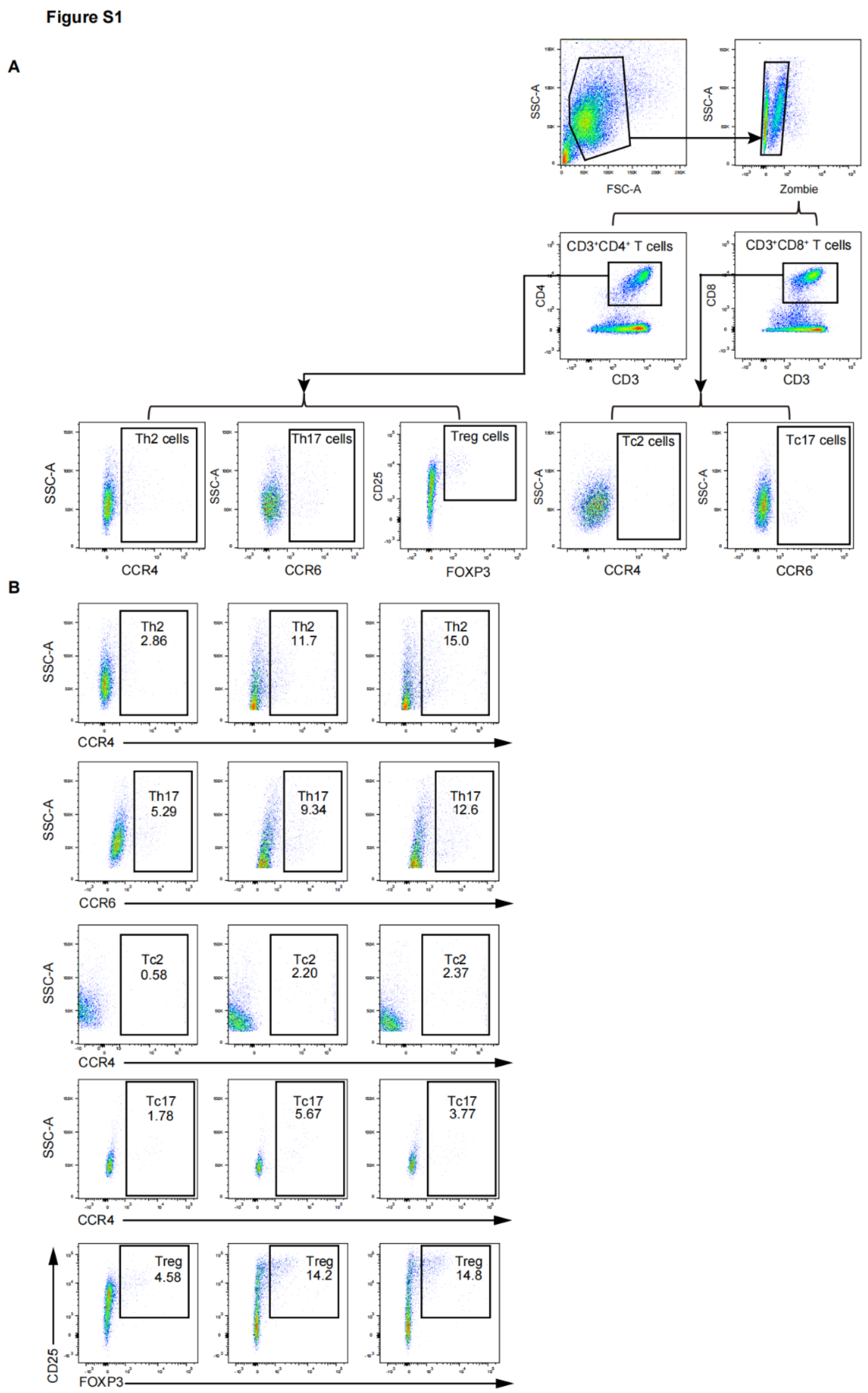

Supplementary Figure S1: The gating strategy and typical diagram of the immunomodulatory role of BM MSCs. A. The gating strategy for detecting different T cell type frequencies. **B.** Flow cytometry to determine the Th2 cells, Th17 cells, Treg cells, Tc2 cells and Tc17 cells.

Figure S2

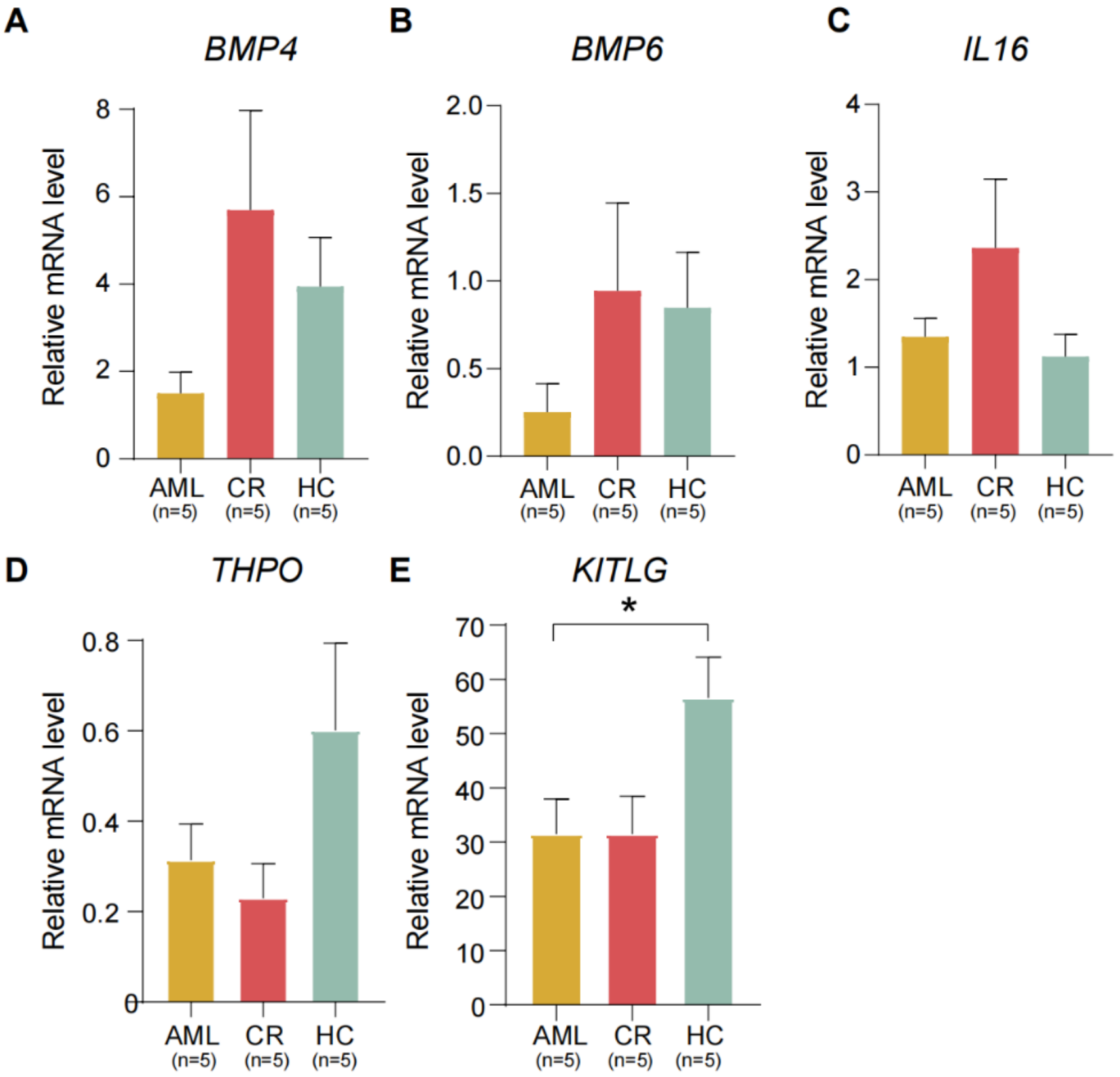

Supplementary Figure S2: The detection of cytokine related gene expression. qRT-PCR to detect the RNA level of *BMP4* **(A)**, *BMP6* **(B)**, *IL-16* **(C)**, *THPO* **(D)**, *KITLG* **(E)**. Each MSC group contains 5 samples (n = 5). *P* values were calculated using one-way ANOVA, and $P < 0.05$ was considered statistically significant. $^*P < 0.05$.

Figure S3

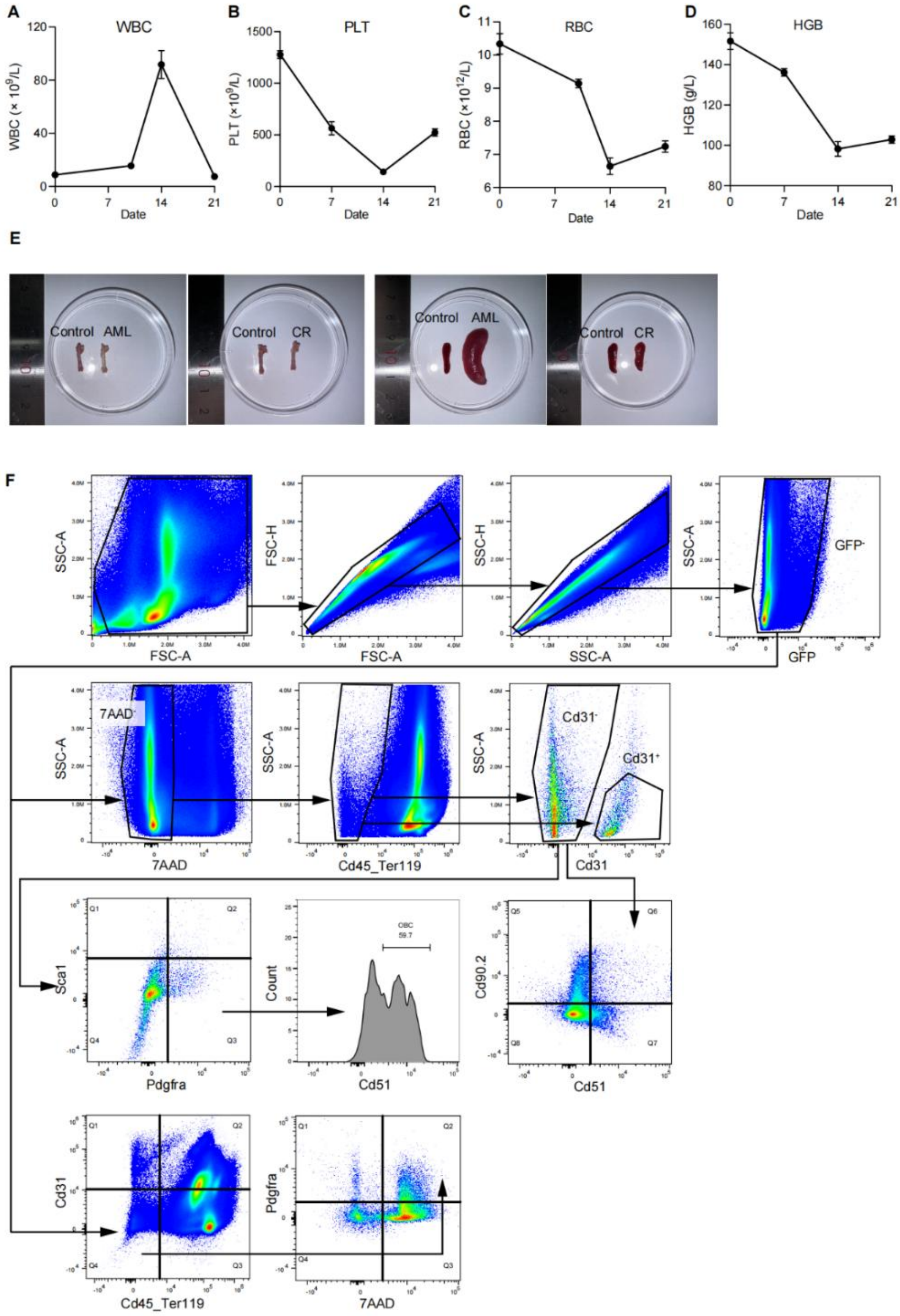

Supplementary Figure S3: The establishment of AML-CR mouse model and gating strategy for flow cytometry. A. Blood routine examination to detect the white blood cell count (WBC). **B.** Blood routine examination to detect the platelet count (PLT). **C.** Blood routine examination to detect the red blood cell (RBC). **D.** Blood routine examination to detect the hemoglobin (HGB). **E.** Representative images of femur and spleen collected from control, AML and AML-CR mice. The AML, CR, Control group consists of 10 mice separately (n = 10). **F.** The gating strategy for flow cytometry analysis.

Figure S4

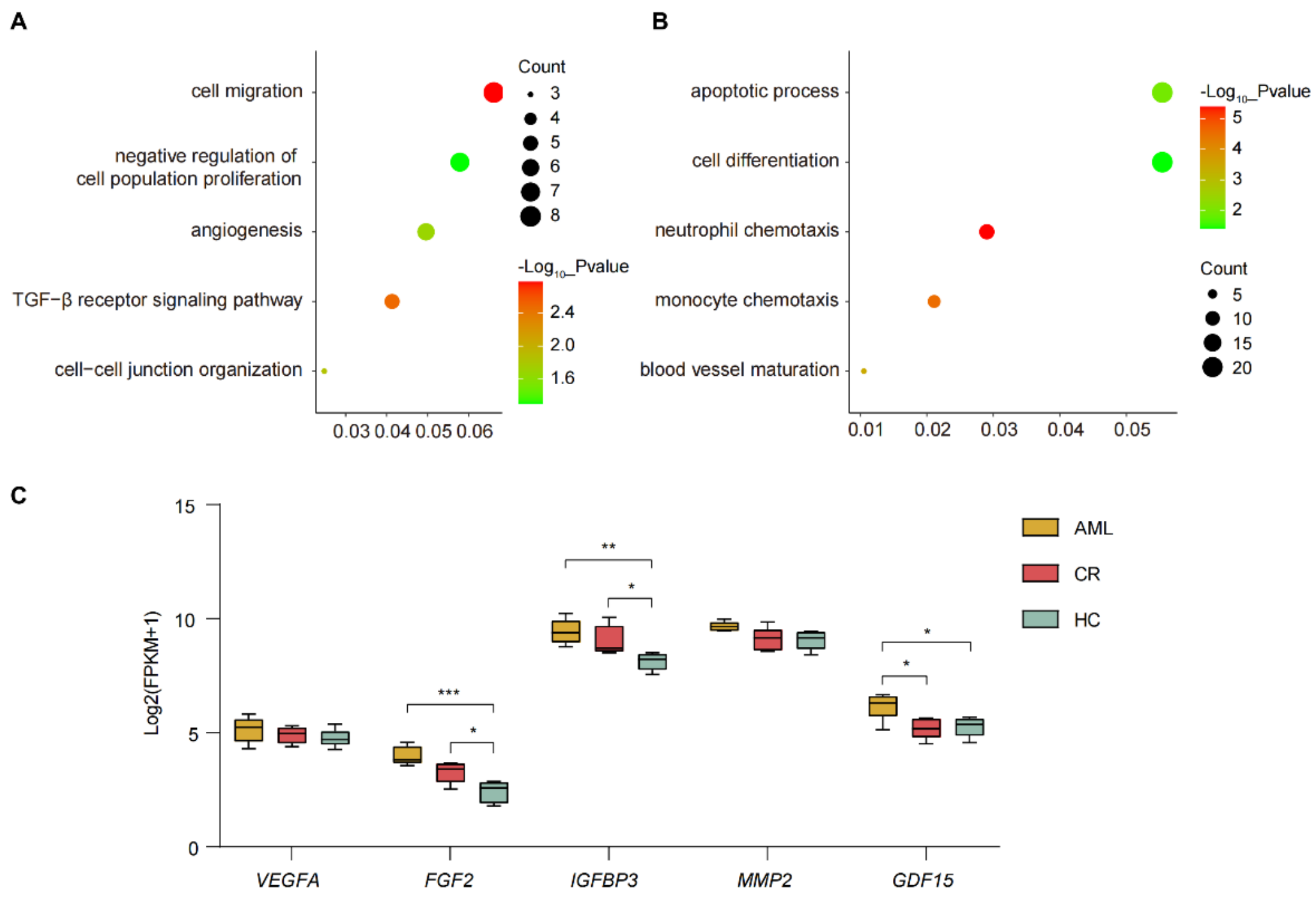

Supplementary Figure S4: Transcriptome wide RNA expression analysis of AML MSCs, CR MSCs and HC MSCs. A. GO analysis of the up-regulated genes in AML MSCs and CR MSCs compared to HC MSCs**. B.** GO analysis of the down-regulated genes in AML MSCs and CR MSCs compared to HC MSCs**. C.** The RNA level of SASP related genes. *P* values were calculated using one-way ANOVA, and $P < 0.05$ was considered statistically significant. $*P < 0.05$, $**P < 0.01$, $***P < 0.001$.

Figure S5

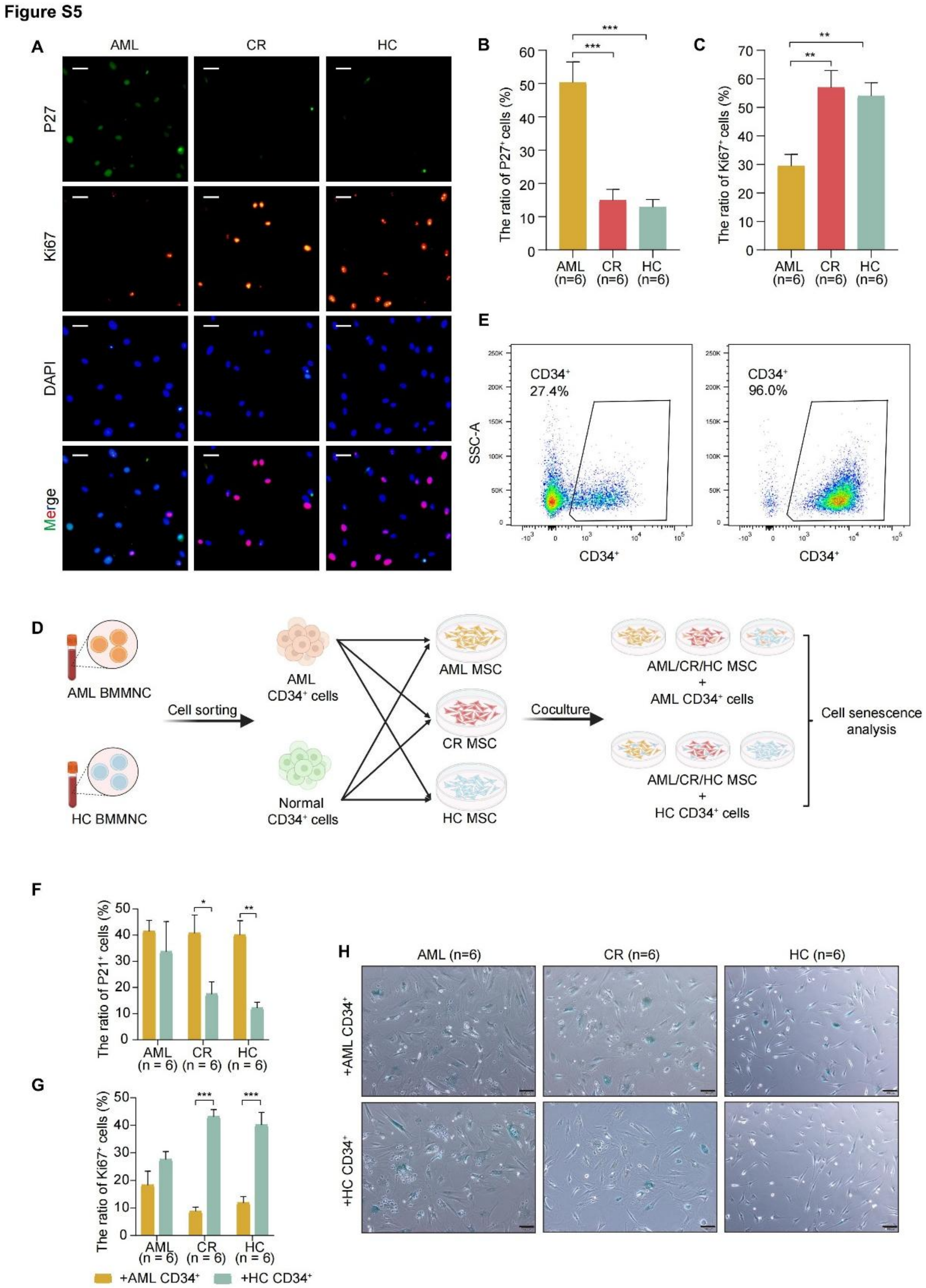

Supplementary Figure S5: The detection of senescent BM MSCs. A. Representative images of immunofluorescence staining of P27 and Ki67 in AML MSCs, CR MSCs and HC MSCs, respectively. Scale bar represents 50 μm. **(B, C)** Statistical analysis of $P27^+$ **(B)** and corresponding $Ki67^+$ **(C)** MSCs in these three groups. Each MSC group contains 6 samples (n = 6), *P* values were calculated using one-way ANOVA. $*P < 0.05$,

$P < 0.01$, ***$P < 0.001$. **D. Schematic diagram of the experimental design for the BM MSC coculture process with CD34$^+$ cells isolated from HC BMMNCs or AML BMMNCs. **E.** Flow cytometry to detect the purity of AML derived CD34$^+$cells before (left) and after (right) sorting. **(F, G)** Statistical analysis of P21$^+$ **(F)** and corresponding Ki67$^+$ **(G)** MSCs from **Figure. 6F**. Each MSC group contains 6 samples (n = 6). *P* values were calculated using two-tailed Student' s t-test, and $P < 0.05$ was considered statistically significant. *$P < 0.05$, **$P < 0.01$, ***$P < 0.001$. **H.** Representative images of SA-β-gal staining in AML MSCs, CR MSCs and HC MSCs after coculture with AML CD34$^+$ cells or HC CD34$^+$ cells. Each MSC group contains 6 samples (n = 6). Scale bar represents 100 μm.

Figure S6

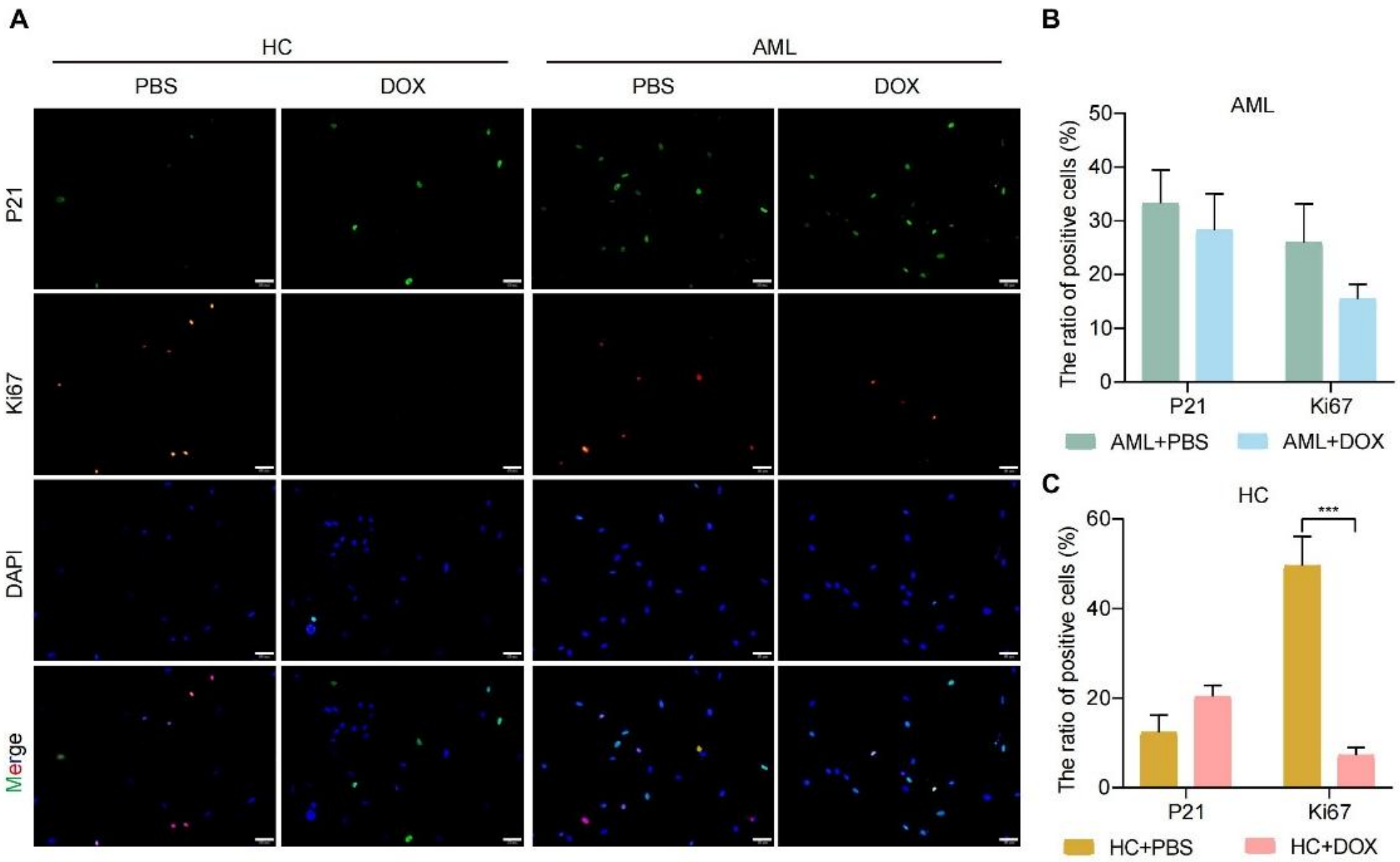

Supplementary Figure S6: The detection of senescent markers in BM MSCs. A. Representative images of immunofluorescence staining of P21 and Ki67 in HC MSCs treated with DOX or PBS respectively. Scale bar represents 50 μm. **B.** Statistical analysis of $P21^+$ and corresponding $Ki67^+$ MSCs in AML MSCs treated with PBS or DOX. **C.** Statistical analysis of $P21^+$ and corresponding $Ki67^+$ MSCs in HC MSCs treated with PBS or DOX. Representative results from six replicates were shown here. P values were calculated using two-tailed Student' s t-test, and $P < 0.05$ was considered statistically significant. *$P < 0.05$, **$P < 0.01$, ***$P < 0.001$.

Figure S7

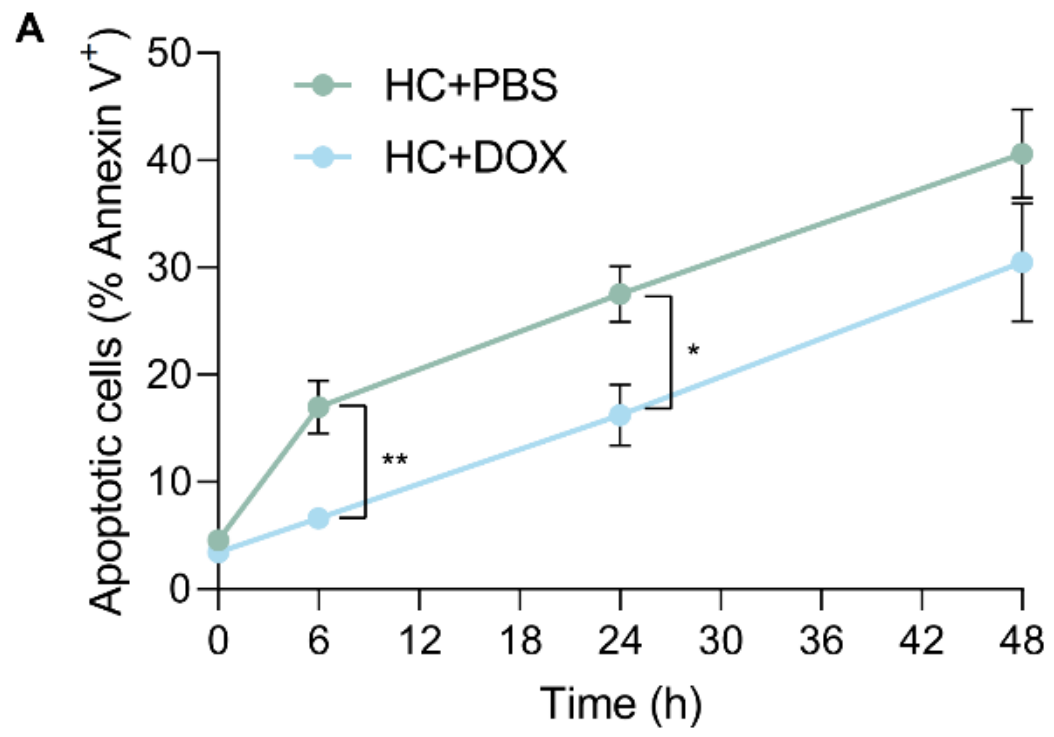

Supplementary Figure S7: The comparison of apoptosis kinetics analysis between HC MSCs and DOX-induced BM MSCs. Representative results from three replicates were shown here. *P* values were calculated using two-tailed Student' s t-test, and $P < 0.05$ was considered statistically significant. $^*P < 0.05$, $^{**}P < 0.01$, $^{***}P < 0.001$.

Supplementary Tables

Supplementary Table S1: The characteristics of patients with AML, patients with CR and HCs

Characteristics	AML (n = 15)	CR (n = 15)	HC (n = 15)	*P* value*	*P* value**	*P* value***
**Physical variables**						
Age (years)[a]	42 (19-65)	43 (23-68)	42 (25-61)	0.6127	0.6152	0.5473
Gender, female vs. male	8/7	8/7	5/10	1.000	0.269	0.269
**BM cell counts**						
BM Blasts (%) [a]	54.67 (15-91)	0.9 (0-2)	/	< 0.0001	0.3917	/
**Blood cell counts**						
WBC count ($\times 10^9$/L) [a]	73.73 (1.41-470.12)	4.32 (1.56-7.75)	6.52 (4.03-9.45)	0.0166	0.0085	0.0181
ANC ($\times 10^9$/L) [a]	22.86 (0.21-185.7)	2.49 (0.74-4.11)	3.95 (1.73-6.6)	0.3203	0.0052	0.3394
Hemoglobin (g/L) [a]	80.33 (47-133)	116.36 (90-138)	143.67 (85-164)	0.0012	0.0051	< 0.0001
Platelet ($\times 10^9$/L) [a]	77.67 (14-290)	181.86 (97-321)	254.00 (185-347)	0.0085	0.0134	< 0.0001

[a] Data are shown as median (range).

* *P* values present the comparison between the CR patients and de novo AML groups. The counts were compared using the Wilcoxon matched-pairs signed rank test. The criterion for statistical significance was $P < 0.05$.

** *P* values present the comparison between CR patients and healthy control. The counts were compared using the Wilcoxon matched-pairs signed rank test. The criterion for statistical significance was $P < 0.05$.

*** *P* values present the comparison between de novo AML patients and healthy control. The counts were compared using the Wilcoxon matched-pairs signed rank test. The criterion for statistical significance was $P < 0.05$.

Abbreviations: AML: acute myeloid leukemia; CR: complete remission; HC: healthy control; BM: bone marrow; WBC: white blood cell; ANC: absolute neutrophil count; ns: not significant

Supplementary Table S2: Clinical characteristics of AML patients

NO.	Sex	Age	BM blasts (%)	$CD34^{+}$ cells (%)	FAB Subtype	WHO subtype	Molecular mutations (at diagnosis)	Cytogenetics (at diagnosis)	Induction Chemotherapy
1-AML	M	19	91	44	M5	AML(FLT3-ITD^{+})	FLT3-ITD=65%; WT1=54.6%	50, XY, +7, +8, +8, +10[18]/46	/
2-AML	F	21	35.5	24.51	M4	AML(DEK-NUP214^{+})	DEK::NUP214=282.8%; WT1=43.4%	6, XX, t (6;9) (p23;q34)[18]/47, idem, +4[1]/46, XX[1]	Ven+DCAG
3-AML	M	22	35	11.6	M4	AML(NUP98::NSD1^{+}, FLT3-ITD^{+})	NUP98::NSD1=217.3%; WT1=17.6%; FLT3-ITD^{+}	47, XY, +8[15]/46, XY[5]	HAA
4-AML	F	29	54	35	M2	AML(AML1-ETO^{+})	AML1::ETO=329.0%; WT1=6.3%	45, X, -X , t(8;21)(q22;q22.1)[19]/45, X, -X, [1]	CAG
5-AML	F	35	25	5	M4	AML(MLL::ELL^{+}, EVI1^{+})	MLL::ELL=4.2%; WT1=17.7%; EVI1= 196.9%	46, XX, t(11;19)(q23;p13.1)[21]/46, XX[2]	Ven+DAC
6-AML	F	37	87	81	M2	AML(CEBPA bZip^{+}, FLT3-ITD^{+})	FLT3-ITD^{+}; WT1/ABL=4.8%; CEBPA bZip^{+}	46, XX[20]	HAA
7-AML	M	41	90	89	M5	AML(MLL-AF6^{+}, EVI1^{+})	MLL-AF6=15.2%; EVI1=25.0%; WT1=6.4%	46, XY, t(6;11)(q27;q23)[18]/46, XY[2]	Ven+DCAG
8-AML	M	43	77.5	50.54	M2	AML(CBFB::MYH11^{+})	CBFB::MYH11=117.1%; WT1=17.3%	46, XY, inv(16)(p13q22)[13]/47, idem, +22[7]	HAA
9-AML	M	46	50	25	M2	AML(MLL-	MLL-PTD=10.6%;	47, XY, +11[13]/46, XY[8]	HAA

						PTD^{+}, IDH2^{+})	WT1=49.1%; IDH2^{+}		
10-AML	F	48	66	72	M2	AML(FLT3-ITD^{+})	FLT3-ITD^{+}; WT1=41.1%	46, XX[10]	/
11-AML	M	55	63.5	58.65	M5	AML(FLT3-ITD^{+})	WT1=9.7%; FLT3-ITD^{+}	46, XY[20]	/
12-AML	F	57	72	43.41	M2	AML(AML1-ETO^{+})	AML1-ETO=390.3%; WT1=17.3%	45,X,-X,t(8;21)(q22;q22)[1]/46, idem, +mar[17]/45, XX, del(9)(q13),-21[1]/56 < 2n>, XX, +11, +12, +13, +13, -15, +18, +18, +20, +20, +21, +21[1]/46, XX[3]	Ven+CAG
13-AML	M	58	58.5	52.89	M2	AML(AML1-ETO^{+})	AML-ETO=247.9%; WT1=5.2%	45, X, -Y, t(8;21), (q22;q22)[17]/46, idem, +4[3]	/
14-AML	F	59	34	16.15	M4	AML(FLT3-ITD^{+})	FLT3-ITD=49%; WT1=55.0%; NPM1=34.97%	46, XX[1]	/
15-AML	M	65	34	21.27	M2	AML(TP53^{+})	TP53^{+}; WT1=2.4%	45, XY, -2, der(5)t(5;15)(q31;q22), add(6)(p23), add(12)(q24), -15, +22[2]/46, idem, +10, +11, -17, -18, +21[5]/47, idem, +10, +11, -17, -18, +21, +22[13]	/

Abbreviations: AML: acute myeloid leukemia; F: Female; M: Male; BM: bone marrow; FAB: French–American–British; WHO: World Health Organization; Ven: Venetoclax; DCAG: Decitabine + Cytosine arabinoside + Aclacinomycin + Granulocyte colony-stimulating factor; HAA: Homoharringtonine + Azacitidine + Cytosine arabinoside; CAG: Cytarabine + Idarubicin + Granulocyte colony-stimulating factor; DAC: Decitabine

Supplementary Table S3: Clinical characteristics of AML-CR patients

NO.	Sex	Age	BM blasts (%)	$CD34^+$ cells (%)	FAB Subtype	WHO subtype	Molecular mutations (at CR)[a]	Induction Chemotherapy	MRD
1-CR	F	23	1.5	0.11	M2	AML(AML1-ETO^+, $NRAS^+$)	WT1=0.21%; AML-ETO^-; FLT3-ITD^-	HAA	Neg
2-CR	F	25	1.5	0.28	M2	AML($NPM1^+$)	$NPM1^-$; $WT1^-$	HAA	Neg
3-CR	F	29	1.5	0.24	M4	AML(CEBPA $bzip^+$)	FLT3-ITD^-; WT1=0.37%;	IA	Neg
4-CR	F	32	0.5	0.36	M2	AML($CEBPA^+$)	$CEBPA^-$, WT1=0.92%	HAA	Neg
5-CR	F	35	0.5	0.09	M2	AML(AML1-ETO^+)	AML-ETO=0.012%	HAA	Neg
6-CR	M	39	0.5	0.14	M2	AML($CEBPA^+$)	WT1=0.34%;	HAA	Neg
7-CR	F	39	0.5	0.12	M4	AML(CBFβ::$MYH11^+$$NRAS^+$)	WT1=0.12%; CBFβ::MYH11=0; FLT3-ITD^-	IDA+Ven	Neg
8-CR	M	39	0.5	0.29	M2	AML(CBFβ-$MYH11^+$, FLT3-ITD^+, KIT-$D816V^+$)	FLT3-ITD^-; WT1=0.14%, CBFβ::MYH11=0.13%	HAA	Neg
9-CR	F	43	0.5	0.11	M4	AML(CBFβ::$MYH11^+$, c-KIT^+)	CBFB::MYH11=0.029%	HAA	Neg
10-CR	M	43	1.0	0.13	M2	AML(CBFβ::$MYH11^+$)	CBFβ-MYH11=0	HAA	Neg
11-CR	M	44	0.5	0.43	M4	AML(CBFβ::$MYH11^+$)	CBFβ::MYH11=0.019%	HAA	Neg

12-CR	M	48	1.5	0.39	M2	AML(CEBPA bZip$^+$, FLT3-ITD$^+$)	WT1=0.33%; FLT3-ITD$^-$	IA	Neg
13-CR	M	59	2.0	0.11	M2	AML(NPM1$^+$, CEBPA$^+$)	WT1=0.10%; NPM1$^-$	IA	Neg
14-CR	F	64	1.0	0.05	M2	AML(AML1-ETO$^+$)	WT1=0.077%; AML-ETO=0.0035%	Ven+Aza	Neg
15-CR	M	68	0	0.14	M4	AML(CBFβ-MYH11$^+$)	CBFβ::MYH11=0.014%	IA	Neg

[a] The normal value of WT1 is “WT1 < 0.6%”.

Abbreviations: CR: complete remission; F: Female; M: Male; Neg: Negative; HAA: Homoharringtonine + Azacitidine + Cytosine arabinoside; IA: Idarubicin + Cytosine arabinoside; IDA: Idarubicin; Ven: Venetoclax; Aza: Azacitidine

Supplementary Table S4: Key Resources Table

**Primers**		
Genes	Forward (5’-3’)	Reverse (5’-3’)
IBSP	GCATTTTGGGAATGGCCTGT	TGAGTCACTACTGCCCTGAAC
SPP1	ACCTGACATCCAGTACCCTGA	ACGGCTGTCCCAATCAGAAG
PLIN2	GATGGCAGAGAACGGTGTGA	TGGCATTGGCAACAATCTGAG
PPARG	TTCCCGCTGACCAAAGCAAA	CAGCCCTGAAAGATGCGGA
BMP2	TTCGGCCTGAAACAGAGACC	CTCCGGGTTGTTTTCCCACT
BMP4	GGAGCTTCCACCACGAAGAA	GGAAGCCCCTTTCCCAATCA
BMP6	TCACGGCCACTAGCAATCTG	ACGTGCACCTCACTCACTTT
CCL3	GCTCTCTGCAACCAGTTCTCT	GGCTTCGCTTGGTTAGGAAGA
CCL4	GCTAGTAGCTGCCTTCTGCT	CCACAAAGTTGCGAGGAAGC
CXCL2	CAGAAAGCTTGTCTCAACCCC	TCAGTTGGATTTGCCATTTTTCAGC
CXCL3	TGTGAATGTAAGGTCCCCCG	TTGGTGCTCCCCTTGTTCAG
CXCL8	ACTCCAAACCTTTCCACCCC	ATGAATTCTCAGCCCTCTTCAAA
KIT	TGCTCTGCTTCTGTACTGCC	GCCTTACATTCAACCGTGCC
KITLG	TCATTCAAGAGCCCAGAACCC	ACTGCTACTGCTGTCATTCCT
IL10	CTCCGAGATGCCTTCAGCAG	TCACATGCGCCTTGATGTCT
IL16	ATCACCAAGGACAGCAGCTC	TTCATTGAGCGTCAGGCAGT
HLA-DQA1	TGGACCTGGAGAGGAAGGAG	CAGCGGTAGAGTTGTAGCGT
OSM	GGTACTGCTCACACAGAGGAC	ATAGGGGTCCAGGAGTCTGC
THPO	CTGCTTCGTGACTCCCATGT	CCCAGAATGTCCTGTGCCTT
TNF	CTTCTCGAACCCCGAGTGAC	TGAGGTACAGGCCCTCTGAT
18S	GTAACCCGTTGAACCCCATT	CCATCCAATCGGTAGTAGCG

Antibodies and Regents		
Name	Source	Identifier
CD34	Biolegend	343505
CD45	Biolegend	368509
CD11b	Biolegend	301306
CD73	Biolegend	344005
CD90	Biolegend	328107
CD105	Biolegend	800509
CD45-APC	Biolegend	103111
Ter119-APC	Biolegend	116211
CD31-PE-CY7	Biolegend	102417
PDGFRα-BV421	Biolegend	135923
SCA1-BV605	Biolegend	108133
CD51-PE	Biolegend	104105
CD90-APC-CY7	Biolegend	140331
7AAD	Biolegend	420403
P21	Cell Signaling Technology	2947T
P27	Cell Signaling Technology	3686T
Ki67	Cell Signaling Technology	9449T
Anti-rabbit IgG	Cell Signaling Technology	4412S
Anti-mouse IgG	Cell Signaling Technology	4409S
Ficoll	TBDscience	LTS1077
DMEM with low glucose	Gibco	11885084
Fetal bovine serum	Gibco	10099141C
Penicillin-streptomycin solution	Gibco	15140122
FGF-basic	Peprotech	100–18B
Dexamethasone	Selleck	S1322
Ascorbic acid	Selleck	S3114
β-glycerophosphate	Selleck	S3620
Alizarin Red S Staining Kit	Beyotime	C0148S
3-isobutyl-1-methylxanthine	Selleck	S5836
Indomethacin	Selleck	S1723
Oil Red O Staining Kit	Beyotime	C0158S
APC Annexin V Apoptosis	BioLegend	640930

Detection Kit with 7-AAD		
CellEvent senescence green flow cytometry assay kit	Invitrogen	C10840
CD34 microbead kit	Miltenyi Biotec	130-046-702
CD3 microbead kit	Miltenyi Biotec	130-097-043
Stem span medium	Stem Cell Technology	09605
Stem cell factor	Peprotech	300-07
Thrombopoietin	Peprotech	300-18
FLT-3 ligand	Peprotech	300-19
RPMI 1640 medium	Gibco	22400-089
MethoCult H4434 Classic	Stem Cell Technology	04434CN
4% Paraformaldehyde	Beyotime	P0099
BSA	Sigma	9048-46-8
Triton X-100	Diamond	9002-93-1
DAPI	Sigma	F6057
β-Galactosidase Staining Kit	Beyotime	C0602
